# Construction of a Bioactive ECM Interface Enables Concurrent Suppression of Foreign Body Reaction, Inflammation, and Promotion of Urethral Regeneration

**DOI:** 10.34133/bmr.0334

**Published:** 2026-03-11

**Authors:** Peihong Han, Xinyu Lei, Shutong Li, Kai Fu, Xiuhong Sun, Rui Zhou, Yuqing Niu

**Affiliations:** ^1^ School of Life and Health Technology, Dongguan University of Technology, Dongguan 523808, China.; ^2^ Provincial Key Laboratory of Research in Structure Birth Defect Disease and Department of Pediatric Surgery, Guangzhou Women and Children’s Medical Center, Guangzhou Medical University, Guangzhou 510623, Guangdong, China.

## Abstract

The development of tissue-engineered urethral grafts (TEUGs) remains challenged by significant hurdles, particularly in overcoming intraurethral stricture. A core issue is the foreign body reaction (FBR) induced by the implant, which impairs the integration of TEUGs with the autologous urethra. This study employed a tissue-engineered cellularization strategy to construct a stable, hydrophilic, and elastic bioactive extracellular matrix (ECM) interface on the scaffold surface. This interface effectively resists FBR and enhances TEUG integration with host tissues. We developed an “autologous” cellularized TEUG by combining rabbit-derived smooth muscle cells and endothelial cells with nanofiber scaffolds in vitro. After seeding, the cells attached to the scaffold, synthesized, and deposited ECM, thereby fine-tuning the scaffold’s biophysical and biochemical properties. Specifically, glycosaminoglycans (GAGs) in the ECM enhanced scaffold hydrophilicity, while collagen and elastin regulated its elasticity. In a rabbit model of full-thickness urethral defect, GAGs in TEUGs induced a pro-regenerative immune response, characterized by up-regulated expression of arginase 1, CCAAT/enhancer-binding protein β, and tissue inhibitor of metalloproteinase 1 genes and down-regulated expression of matrix metalloproteinase 9 and interleukin-12 genes. This M2 macrophage-dominated gene expression profile further activated the Th2 signaling pathway, promoting the reconstruction of damaged vascular networks, the ordered proliferation of new tissues, the replacement of original ECM, load transmission in new tissues, and the maturation of functional structures. This study provides a simple yet effective strategy to enhance the patency, urine transport capacity, synchronous contraction, and directional contractile function of TEUGs by engineering a bioactive ECM interface endowed with anti-inflammatory and anti-FBR properties.

## Introduction

Urethral injuries, whether due to congenital defects, trauma, or disease, are a prevalent and costly health issue [1]. In the United States alone, the annual cost of treating these injuries approaches $200 million, with urethral reconstruction surgery alone accounting for over $10 million annually [2]. Clinically, urethral injuries are typically repaired using flaps or autologous urethral grafts [3]. However, these approaches are limited by donor tissue scarcity, high surgical costs, and donor-site morbidity. To address these challenges, tissue-engineered urethral grafts (TEUGs) have emerged as a promising solution [4]. Despite extensive preclinical and clinical efforts [5–12], TEUGs often fail in cases involving long-segment strictures (>2 cm), tubular grafts, grafts placed on unhealthy residual urethral beds, or penile stricture grafts [1]. A major contributor to this failure is the foreign body reaction (FBR) triggered by the implant, which critically impairs integration between the graft and host tissue.

Upon implantation, biomaterials are immediately recognized as foreign by the host immune system. This initiates a cascade of events at the implant–tissue interface known as FBR [13]. The response is characterized by intense inflammation, formation of foreign body giant cells, fibrosis, and eventual collagen encapsulation. Although FBR serves as a protective mechanism to isolate the implant, it severely hinders functional integration with native tissue [14]. In the context of TEUGs, FBR is especially detrimental. Fibrosis and chronic inflammation can lead to recurrent urethral stricture and, in severe cases, complete obstruction. This may result in dysuria, urinary retention, and other complications [1]. Therefore, mitigating FBR is essential for improving the biocompatibility and long-term success of TEUGs. Previous anti-FBR strategies include hydrogel coatings [15,16], bionic patches [17,18], and extracellular matrix (ECM) biomimicry of scaffold components [12,19,20]. However, these approaches face significant limitations, such as the lack of durable materials and the fact that most scaffolds have not received U.S. Food and Drug Administration approval, creating a major barrier to clinical translation. To circumvent reliance on synthetic scaffolds, researchers have explored cell-only strategies using cell sheet technology. In this approach, cell sheets are first cultured around a mandrel to form a stable tubular structure. They are then perfused to promote wall maturation and epithelialization. Yet, this process requires 4 to 7 weeks of culture time, which limits its utility in both emergency and elective urethral reconstruction surgeries [4,5,10].

Current anti-FBR strategies for TEUGs, such as hydrogel coatings, ECM mimicry, and cell sheet technology, fail to simultaneously achieve effective FBR suppression, mechanical compatibility, and clinical feasibility [4,12,17]. Hydrogel coatings often lack structural integrity, while cell sheet methods suffer from prolonged manufacturing timelines [4,16]. Furthermore, most studies neglect the coordinated regulation of the immune microenvironment and tissue mechanics, both of which are critical for successful graft integration [20,21]. Notably, our strategy reduces the functional TEUG fabrication timeline to 2 weeks. This is significantly shorter than the 4 to 7 weeks typically reported in prior studies. Importantly, this accelerated protocol does not compromise structural integrity or immunomodulatory capacity, thereby addressing a key bottleneck in clinical translation. In contrast to existing approaches, our design integrates a mechanically robust electrospun scaffold with a bioactive interface. This interface is formed by layer-specific secretion of ECM from smooth muscle cells (SMCs) on the outer surface and urethral epithelial cells (UECs) on the luminal side. The dual-layer architecture overcomes the limitations of single-modification or scaffold-free strategies. It enables simultaneous control over immune modulation and mechanical adaptation, representing a novel advance in TEUG development.

**Fig. 1.**
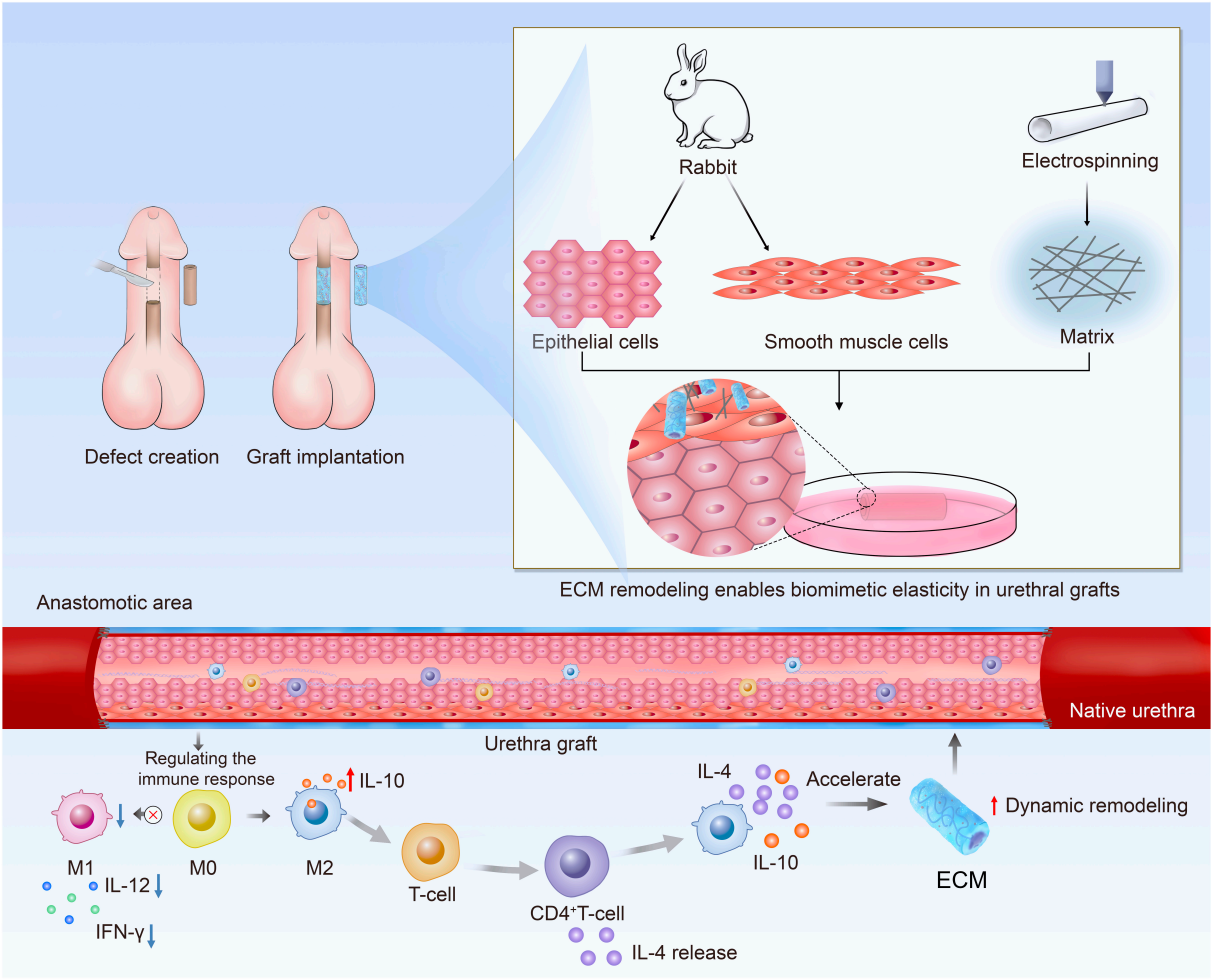
Schematic diagram of constructing a bioactive ECM interface on TEUG based on autologous urethral-derived ECs and SMCs, and its potential in enabling simultaneous suppression of foreign body reaction, inflammation, and promotion of urethral regeneration during full-thickness urethral repair.

To address FBR and inflammation during implantation, we combined electrospinning with cell-based tissue engineering. Rabbit-derived SMCs and UECs were selectively seeded onto the outer and inner surfaces, respectively, of a biodegradable nanofibrous scaffold, generating a TEUG with “autologous-like” features. After implantation, these cells adhered to the scaffold and deposited a functional ECM interface that played 3 pivotal roles in urethral regeneration. First, glycosaminoglycans (GAGs) in the ECM promoted a pro-regenerative gene expression profile. They up-regulated arginase 1 (*ARG1*), CCAAT/enhancer-binding protein β (*CEBPB*), and tissue inhibitor of metalloproteinase 1 (*TIMP1*), while down-regulating matrix metalloproteinase 9 (MMP9) and interleukin-12 (IL-12). This shifted macrophages toward the anti-inflammatory M2 phenotype. The effect was further amplified through an IL-4/IL-10-positive feedback loop, which activated Th2-mediated immune responses and effectively suppressed FBR and inflammation. Second, ECM components, such as type I and III collagens and elastin, fine-tuned the scaffold’s elastic modulus. This allowed the mechanical properties to closely match those of native urethral tissue. Meanwhile, GAGs enhanced hydrophilicity, fostering a favorable microenvironment for cell adhesion and migration. Third, the immunomodulatory microenvironment facilitated recruitment and directed differentiation of endogenous stem cells. This ultimately drove the regeneration of functional urethral epithelium and smooth muscle layers (Fig. 1).

## Materials and Methods

### Experimental design

This study aims to explore the feasibility of generating TEUGs with urethral biological properties by obtaining autologous urethral SMCs and endothelial cells (ECs) from patients, expanding them in vitro, and then seeding them onto biodegradable scaffolds. The research also seeks to validate whether the seeded cells serve as the critical foundation for the formation of new urethral function. Although clinical and animal model studies have confirmed the successful reconstruction of functional new urethras using TEUGs, research in this field remains relatively scant. Initially, we prepared nanofiber tubes that match the internal diameter and wall thickness of the urethra of New Zealand white rabbits. Subsequently, urethral SMCs and ECs, isolated and expanded in vitro from young rabbits, were seeded onto the outer and inner surfaces of the nanofiber tubes, respectively, followed by a 7-day coculture period in vitro to form TEUGs containing SMCs and ECs. To investigate the early stages of TEUG–host interaction (i.e., 14, 30, and 60 days post-implantation) and the process of TEUGs maturing into functional live urethras, we evaluated the mechanical properties (including suture retention strength, circumferential and longitudinal elastic mechanical properties, and intraurethral pressure during urination) and regenerative capabilities of TEUGs using an autologous urethral implantation model in young rabbits (*n* = 3). During this process, we focused on observing the interaction between the seeded SMCs and ECs and the host-derived endogenous cells.

### Preparation of biodegradable PLA/gelatin nanofiber tubular scaffolds

To prepare biodegradable polylactic acid (PLA)/gelatin nanofiber tubular scaffolds, we employed the solution spinning technique based on previous research methods [22] and conducted the following operations. Briefly, 0.05 g of left-handed PLA (*M*_n_ = 40,000 Da, 94829, Sigma-Aldrich) and 0.1667 g of gelatin (*M*_w_ ≤ 6,500, 920029, Sigma-Aldrich) were dissolved in 10 ml of a 3:1 (v/v) hexafluoroisopropanol (H10750, Aladdin) and trifluoroethylene (TFEA, T109512, Aladdin) mixed solvent at room temperature under magnetic stirring to form a homogeneous spinning solution. The solution was loaded into a syringe pump and electrospun at a flow rate of 120 μl/min. A 2.7-mm-diameter rotating collector rod (390 rpm) was positioned 25 cm from the nozzle. High and low voltage were set to 12 and −1.6 kV, respectively. Electrospinning was performed for 48 h under controlled conditions (18 to 25 °C, 45% relative humidity). The resulting tubular scaffold was carefully peeled off, trimmed to the desired length, and vacuum-dried at room temperature for 24 h to remove residual organic solvents. Finally, scaffolds were sterilized with 12 kGy ^60^Co γ-radiation for 30 min and stored dry for later use.

### In vitro construction of TEUG

Within a laminar flow hood, sterile PLA/gelatin nanofiber scaffolds were longitudinally sectioned to obtain nanofiber sheets, which were then placed in corresponding 3.5-cm^2^ polystyrene culture dishes (353001, Falcon) with the outer surface of the nanofiber membranes facing upwards. The dissociated SMC suspension was seeded onto the PLA/gelatin nanofiber sheets at a concentration of 1 × 10^7^ cells/ml. The cell-seeded polymer nanofiber membranes were maintained in a 37 °C incubator with 5% CO₂ and 95% humidity for 12 h to allow cell adhesion to the membranes. Subsequently, 2 ml of culture medium was added to each dish, and the cells were incubated for 3 days. After this incubation period, the other side of the membrane was turned upwards, and 1 ml of an ECs suspension (1 × 10^7^ cells/ml) in Dulbecco’s Modified Eagle Medium containing 10% fetal bovine serum was seeded onto it. The membranes were again placed in the incubator for 12 h to allow cell adhesion, followed by the addition of 2 ml of culture medium and another 3 days of incubation. The membranes seeded with SMCs and ECs were then wrapped around a sterile stainless-steel mold, and a 20-μl aliquot of sterile surgical adhesive (1469SB, 3M) was applied along the original incision to seal the wound and form the TEUG.

### Animal experiment subjects and surgical procedures

This experimental protocol has obtained approval from the Experimental Animal Ethics Committee of the Guangdong Medical Laboratory Animal Center (Approval Number: C202303-3). The experiment utilized 24 healthy male New Zealand White rabbits aged 4 months, with a body weight of (2.5 ± 0.1) kg. These rabbits were randomly divided into 3 groups: the autologous transplantation group (using rabbit autologous urethral tissue), the TEUG group, and the pure scaffold group, with 9 animals in each group.

The experimental animals were anesthetized via intraperitoneal injection of pentobarbital sodium at a dosage of 45 mg/kg body weight (P3761, Sigma-Aldrich). Subsequently, a 2.2-cm-long segment of intact anterior urethral tissue was excised between the penis and bulbous urethra. Urethral defects were reconstructed as follows: the TEUG group was implanted with prepared TEUG material, the pure scaffold group was implanted with a scaffold, and the autologous transplantation group was repaired using autologous urethral tissue.

Post-surgery, all animals were housed individually in cages to ensure free access to food and water.

### Cyclic loading-unloading test

A dynamic mechanical analyzer (DMA, Q800, TA Instruments) was employed to conduct uniaxial tensile cyclic loading-unloading tests on TEUGs constructed in vitro, native urethral tissues, and scaffold materials. The samples were cut into rectangular shapes, oriented along their circumferential and longitudinal directions, respectively, and then stored and pre-immersed in phosphate-buffered saline (PBS) 1× solution at 37 ± 0.5 °C. Three replicate samples were prepared for each sample in each tensile direction. The thickness and width of the samples were precisely measured using a micrometer [23].

During the testing process, tensile loads were applied at a constant rate of 1 mm min^−1^, with maximum strains set at 60% for TEUGs and native urethral tissues, and adjusted to 25% for scaffold materials due to their inferior elasticity. All samples were tested at a working temperature of 37 °C within the DMA, and each sample underwent 10 loading/unloading cycles.

### Scanning electron microscopy and transmission electron microscopy examination

In this study, the TEUGs constructed in vitro were initially fixed in electron microscope fixative (G1102, ServiceBio) for 2 h at room temperature in the dark. Subsequently, the samples underwent gradient ethanol dehydration (concentrations of 70%, 80%, 90%, and 100% sequentially). Following dehydration, the samples were freeze-dried [24]. Surface morphologies of the samples were then observed using a high-resolution field emission scanning electron microscope (Regulus 8100, HITACHI, Japan) at an accelerating voltage of 3 kV. Prior to scanning electron microscopy (SEM) observation, each sample was mounted on an aluminum stub and sputter-coated with gold. Notably, PLA/gelatin nanofiber tubular scaffold samples did not require fixation or dehydration prior to observation.

For transmission electron microscopy (TEM), the TEUGs retrieved 30 days post-implantation and fixed with electron microscope fixative were embedded in imported epoxy resin (GP2001, ServiceBio). Ultra-thin sections of 60 nm thickness were prepared using a Leica EM UC7 ultra-microtome. After picking up the sections with copper grids and staining with osmium tetroxide (GP2004, ServiceBio), the samples were visualized using a HITACHI HT 780 TEM at an accelerating voltage of 80 kV, with a resolution of 1.0 nm and a 200× high-contrast mode. Semi-transparent ECM areas undergoing deposition were identified, marked via screen capture, and images of the ECM and cellular morphologies were acquired in a 1,500× low-magnification mode.

### Functional diagnosis

At various time points, animals were anesthetized and placed supine on an examination table. A sterile, disposable 6F veterinary urinary catheter (Model No. 55588, approved by Su Medical Device Registration 20142140677, Yangzhou Dada Medical Devices Co., Ltd.) was inserted into the urethra until the balloon was fully positioned at the urethral orifice. The balloon was inflated with 2 ml of sterile distilled water. An appropriate amount of diluted urografin contrast agent (BS334, Biosharp) was slowly injected through the catheter. After catheter removal, the bladder was gently massaged, and voiding cystourethrography images were obtained using a digital x-ray fluoroscopy system (Siemens, Luminos dRF).

During voiding, the BL420 biological signal acquisition system was activated, entering the biological function experiment software to select the urological system experimental module. One channel was connected to a urethral transducer to record intra-urethral pressure waveforms during voiding. After recording, sterile saline (0.9% NaCl, G4702, Servicebio) was infused into the bladder through the catheter. Subsequently, a solution containing norepinephrine (NA, Approval No. H42021301, Yanda Pharmaceutical, China Co., Ltd.) in saline was used along the catheter to stimulate the anterior urethra. Post-stimulation urethral physiological function was recorded using the BL420 system after catheter removal.

Following the in vivo assessment of urethral physiological function, animals were euthanized, and grafts were harvested. Tissue samples were sutured at both ends and connected to the organ bath system BL420. Samples were submerged in Hank’s Balanced Salt Solution pre-equilibrated with a mixture of 95% oxygen and 5% carbon dioxide at 37 °C for 20 min. After positioning the tissue sample perpendicular to the tension sensor, the preload was adjusted to 1.0 g, followed by a 10-min stabilization period. An electrical stimulator was placed externally on the graft to induce contraction via magnetic field stimulation. Tissue viability was assessed starting with continuous 0.5-mV stimuli. The pattern involved serial stimulation at 1.0 Hz frequency, for 3 pulses, with each pulse having an appropriate duration. All tests were completed within 30 min of tissue extraction.

### Histological analysis

At the predetermined time point, after being euthanized with pentobarbital sodium, the experimental rabbits were immediately sacrificed, and new urethral samples from the surgical area were collected from each group. The newly collected urethral samples and the TEUG samples constructed by in vitro culture in advance were respectively placed in 1.5-ml centrifuge tubes. Using the same method, they were fixed with a 4% paraformaldehyde solution (Beyotime, P0099) at 4 °C for 18 h, and then thoroughly rinsed with distilled water. After the rinsing was completed, the 2 types of samples were respectively dehydrated in graded alcohol solutions and then separately embedded in paraffin (Sigma-Aldrich, 76242) for sectioning.

Standard methods were employed to cut the samples into 5-μm-thick sections, which were stained using hematoxylin and eosin (HE, GP1031, Servicebio), Masson’s trichrome (MTC, G1006, Servicebio), Verhoeff–Van Gieson (VVG, GP1035, Servicebio), Sirius red (SR, GP2088, Servicebio), and Alcian blue (AB, G1049, Servicebio) staining kit. The samples were scanned and imaged using a microscope attached to a Pannoramic MIDI slide digital scanner, and representative fields of view were selected and recorded using Caseviewer software (version 2.3). For SR-stained samples, after locating the target area using the Leica STELLARIS 5 super-resolution white light laser confocal microscope, switch to polarized light mode for observation. Different types of collagen fibers exhibit characteristic color and brightness differences under polarized light, facilitating their differentiation and analysis.

For immunofluorescence analysis, paraffin sections were first subjected to heat-induced antigen retrieval in antigen retrieval solution (G1201, Servicebio) for 15 min, followed by ethanol gradient dehydration and xylene dewaxing (G1128, Servicebio) for rehydration. The rehydrated tissue sections were circled and marked using an immunofluorescence pen. According to the manufacturer’s instructions, the sections were incubated overnight at 4 °C with the following primary antibodies diluted 200 times: mouse anti-cytokeratin 19 (CK19, GB15198, Servicebio) monoclonal antibody, rabbit anti-alpha smooth muscle actin (α-SMA, GB111364, Servicebio) polyclonal antibody, mouse anti-CD44 (GB14037, Servicebio) monoclonal antibody, rabbit anti-calponin (GB11954, Servicebio) polyclonal antibody, mouse anti-Ki67 (GB14102, Servicebio) monoclonal antibody, rabbit anti-CK5 (GB111246, Servicebio) polyclonal antibody, rabbit anti-CD206 (GB113497, Servicebio) polyclonal antibody, sheep anti-hyaluronic acid (HA, 5029-9990, Bio-Rad) polyclonal antibody, or mouse anti-CD86 (ab220188, abcam) monoclonal antibody.

After incubation, Cy3-labeled donkey anti-rabbit secondary antibody (GB21403, Servicebio), Cy5-labeled goat anti-rabbit secondary antibody (GB27303, Servicebio), Rabbit Anti-Sheep IgG H&L Alexa Fluor 555 (ab150182, Abcam), Rabbit Anti-Sheep IgG H&L Alexa Fluor 488 (ab150181, Abcam), and Alexa Fluor 488-labeled goat anti-mouse secondary antibody (GB25301, Servicebio) were added. The samples were then scanned and imaged using a microscope attached to a Pannoramic MIDI slide digital scanner again, and representative fields of view were selected and recorded using Caseviewer software (version 2.3).

The FiJi ImageJ software (version Java 6) was employed for the relative quantitative analysis of target components—including collagen, collagen I, collagen III, elastin, GAGs, and specific protein expression—across MTC, SR, EVG, AB, and immunofluorescence staining. Briefly, stained images were imported into the software, and the scale was calibrated using *Analyze > Set Scale*. Images were then converted to grayscale, contrast-adjusted, and background-subtracted via *Process > Subtract Background*. Thresholds were manually set, and for color images, *Color Deconvolution* was applied to isolate specific channels. The area of positive regions was measured, and the percentage of positive area relative to the total area was calculated to quantify component abundance.

### Quantitative RT-PCR analysis

The expression levels of target marker genes TNF-α, IL-12, MMP9, IL-10, *TIMP1*, vascular endothelial growth factor (VEGF), resistin-like molecule α, *ARG1*, and *CEBPB* were detected by quantitative RT-PCR to determine the polarization state of recruited host monocytes. Total RNA was extracted using an RNA extraction kit (G3689, Servicebio) strictly according to the manufacturer’s protocol. The concentration of extracted RNA was measured using a Nanodrop 2000 spectrophotometer (ND2000, Thermo Fisher Scientific) to ensure the quality and quantity of RNA met the requirements for subsequent experiments. RNA was reverse transcribed into complementary DNA (cDNA) using AccuPower CycleScript RT premix (K2044, Bioneer) following the manufacturer’s instructions. Real-time PCR amplification was performed using SYBR green fluorescent dye (Applied Biosystems) and specific primers (Cosmo Genetech). PCR reactions were conducted on the StepOne real-time PCR system (V2.3, Applied Biosystems) with a 40-cycle amplification program. The amplification conditions included an initial denaturation stage at 95 °C for 10 min, followed by 40 cycles of 95 °C for 15 s and 60 °C for 1 min. Glyceraldehyde 3-phosphate dehydrogenase (GAPDH) was used as the housekeeping reference gene for normalization. The relative expression levels of target genes were calculated using the 2^−ΔΔCt^ method. The primer sequences for all target genes and GAPDH are provided in Table S1.

### Enzyme-linked immunosorbent assay

Following the manufacturer’s instructions, enzyme-linked immunosorbent assay (ELISA) kits (specific models: IL-10, RBDL00031; IL-12, RPAB0053; IL-4, RBDL00038; interferon-gamma [IFN-γ], AGIM0378, all sourced from AssayGenie) were employed to measure the cytokine levels of IL-10, IL-12, IL-4, and IFN-γ within TEUGs, pure scaffolds, and autologous grafts and in plasma.

To evaluate local cytokine concentrations, samples of TEUGs, scaffolds, and autologous grafts bridging urethral defects were collected 7 days post-implantation of each therapeutic material. Residual blood was removed by rinsing with ice-cold PBS, and tissues (50 to 100 mg) were blotted dry, weighed, and minced into 1- to 2-mm^3^ pieces. Ice-cold RIPA lysis buffer (Cat. No. R0278, Sigma-Aldrich; 150 mM NaCl, 1% IGEPAL CA-630, 0.5% sodium deoxycholate, 0.1% SDS, and 50 mM Tris, pH 8.0) was added at a ratio of 1:5 to 10 (w/v), supplemented with protease (10 μl/ml, P2714) and phosphatase (10 μl/ml, P0044) inhibitors to prevent protein degradation. After homogenization, homogenates were digested with Type I collagenase (1 mg/ml) and DNase I (0.1 mg/ml) at 37 °C for 30 min to disrupt ECM and reduce viscosity. Samples were incubated on ice for 30 to 60 min with gentle vortexing every 10 to 15 min, then centrifuged at 12,000 to 14,000 ×*g* for 15 to 20 min at 4 °C. The supernatant (total protein extract) was transferred to a new sterile tube for downstream use.

For the detection of plasma cytokine levels, serum samples were isolated from young rabbits receiving different treatment regimens and appropriately diluted for analysis.

### Statistical analysis

Statistical analyses were conducted using GraphPad Prism software package (version 10.0, GraphPad Software, 2023). For single comparisons between 2 independent datasets, an unpaired Student *t* test was employed. One-way analysis of variance (ANOVA) was utilized for multiple comparisons across a single variable, while 2-way ANOVA was applied for comparisons involving 2 variables. Post hoc analyses were conducted using Tukey’s test following all ANOVA procedures. Data are presented as mean values ± standard deviation (SD). Significance was accepted when *P* < 0.05.

## Results

### Development and characterization of a bioactive ECM interface in TEUGs in vitro

**Fig. 2.**
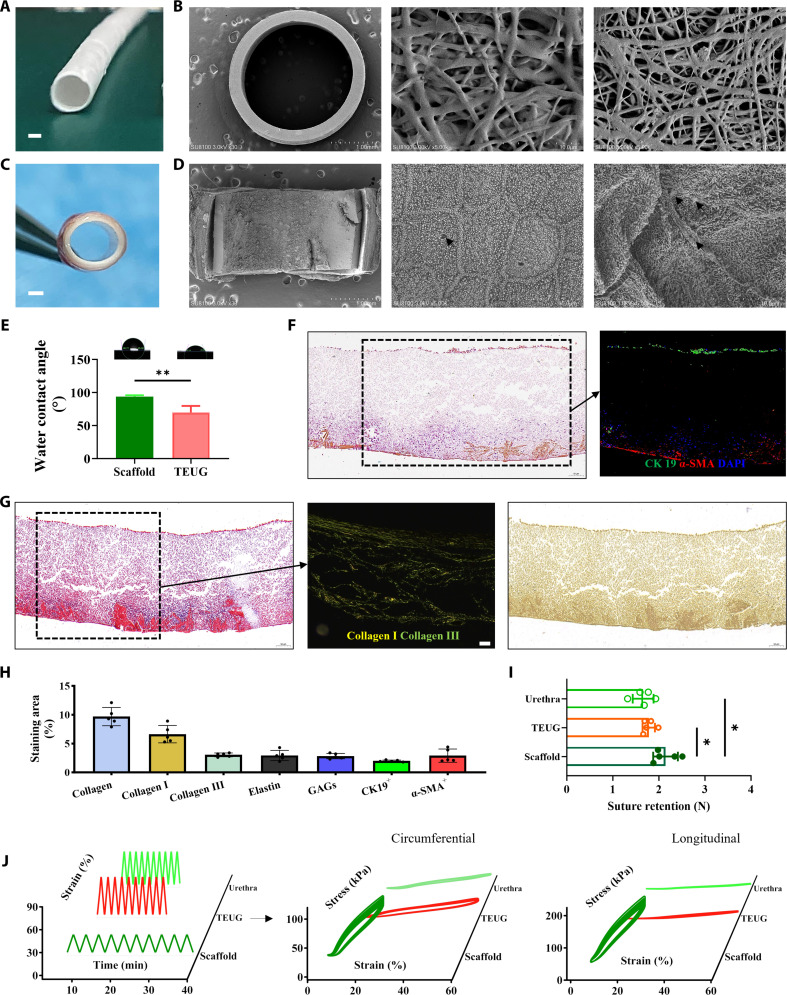
Preparation and characterization of TEUGs with bioactive ECM interface in vitro. (A and C) Digital images of the biodegradable PLA/gelatin nanofiber tubular scaffold (A) and the TEUG after cell seeding with ECs and SMCs (C). (B and D) SEM images showing cross-sections and inner and outer surfaces of the scaffold (B) and TEUG (D). Black arrows indicate uncovered scaffold nanofibers. (E) Water contact angle analysis of scaffolds and TEUGs (*n* = 3). (F) AB staining (left panel) and immunostaining (right panel) for CK-19 (EC marker) and α-SMA (SMC marker) in the mid-cross-section of TEUG. (G) MTC, SR (polarized light), and VVG staining of serial cross-sections of the TEUG. (H) Quantification of CK-19^+^ ECs and α-SMA^+^ SMCs, as well as ECM components including collagen, collagen I, collagen III, elastin, and GAGs in TEUG. Data are mean ± SD; *n* = 5. (I) Suture retention strength of native urethra, TEUG, and scaffold. Data are mean ± SD; *n* = 5; *P < 0.05. (J) Elastic response testing of native urethra, TEUG, and scaffold. Left: Representative 3D waterfall plot of strain over time. Middle and right: 3D plots of circumferential and longitudinal stress-strain responses, respectively. Scale bars: (A and C) 1 mm and (G) 50 μm.

To develop a bioactive ECM interface within TEUGs, we fabricated nanofibrous tubular scaffolds using PLA and gelatin via electrospinning, with an inner diameter of approximately 2.8 mm and wall thickness of ~290 μm (Fig. 2A and B). SMCs and ECs were isolated from the urinary tract of experimental rabbits, expanded in vitro, and then seeded onto the outer and inner surfaces of the scaffold, respectively. After 7 days of culture, a cell-seeded TEUG construct was successfully established (Fig. 2C and Fig. S1A and B).

Microscopic observation revealed that the ECs on the inner surface exhibited a flat morphology similar to that of native urethral epithelium, while the spindle-shaped SMCs on the outer surface displayed interwoven myofilaments, closely resembling their natural counterparts (Fig. 2D and Fig. S2B). Notably, both cell types arranged themselves in an organized manner and actively secreted functional ECM components, effectively replicating the complex luminal architecture of the native urethra (Fig. 2D and Fig. S2B).

Further characterization showed that the hydrophilicity of the TEUG was significantly enhanced compared to the pristine scaffold, as evidenced by reduced water contact angles on the outer wall (Fig. 2E). AB staining confirmed the presence of GAGs within the deposited ECM (blue-violet staining), which are critical for maintaining hydration and modulating immune responses. Immunostaining for CK19 and α-SMA further verified the spatial distribution of ECs and SMCs, aligning precisely with GAG localization (Fig. 2F and Fig. S3). Additionally, the TEUG wall was rich in collagen I and III, which formed an interconnected fibrous network and synergized with elastin to provide structural integrity (Fig. 2G and Fig. S4).

The integration of urethral epithelium-like mucosal tissue, smooth muscle-like layers, and their secreted ECM created a functional framework with biophysical and biochemical properties closely mimicking those of the native urethra (Fig. 2H), consistent with previous observations in human urethral anatomy [1]. This engineered structure significantly improved the biomechanical performance of the TEUG. The suture retention strength of the TEUG was not only markedly superior to that of the pure scaffold but also approached that of native urethral tissue (Fig. 2I). Moreover, both axial and longitudinal elasticity were significantly enhanced compared to the scaffold alone (Fig. 2J and Fig. S5), largely due to the secretion of elastin-rich ECM by the seeded cells—components essential for dynamic load-bearing capacity and tissue deformability [1]. In contrast, the pure scaffold, composed primarily of PLA fibers with only 25% gelatin content, failed to recapitulate the complex elastic characteristics of native urethral tissue (Fig. 2J and Fig. S5). These results demonstrate that the incorporation of SMCs and ECs enables the formation of a structurally and functionally bioactive ECM interface.

### Functional load transmission in ECM interface-mediated neo-tissue reconstruction

**Fig. 3.**
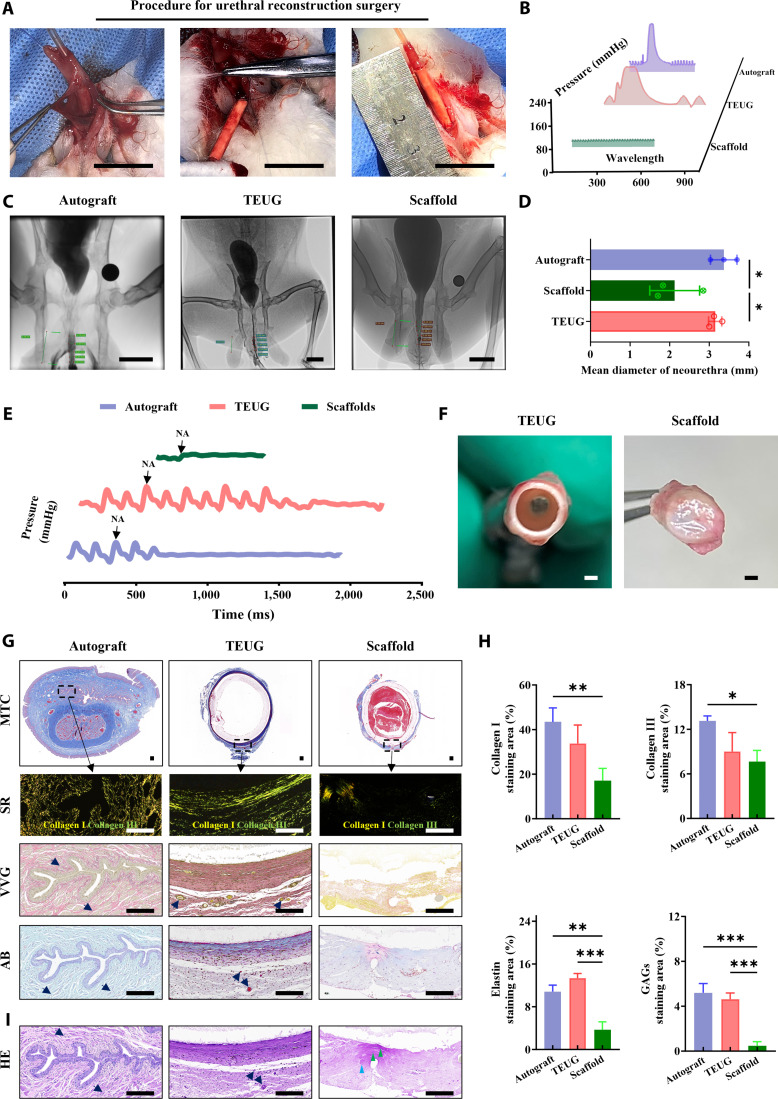
TEUG with a bioactive ECM interface-repaired neo-tissue demonstrates stable load transmission at the urethral defect site. (A) A 2.2-cm urethral defect was created in male New Zealand white rabbits to model long-segment irreversible urethral injury. End-to-end anastomosis was performed using TEUG as an interpositional graft for reconstruction. Assessments were conducted 60 days post-surgery. (B) Voiding urethral pressure was measured using the BL420 biological signal acquisition system. (C) Voiding function and lumen patency were evaluated by cystourethrography. (D) Relative quantification of average neo-urethra diameter. Data are presented as mean ± SD (*n* = 3); *P < 0.05. (E) Urethral tissue response curves to NA stimulation. (F) Macroscopic appearance of cross-sections from retrieved TEUG and scaffold samples. (G) MTC, SR (under polarized light), VVG, and AB staining of mid-cross-sections from retrieved urethral tissues. Black arrow: microvessel; green triangle: lymphocyte infiltration; blue triangle: local tissue edema. (H) Quantitative analysis of collagen I, collagen III, elastin, and GAG content in each group. Data are mean ± SD; *n* = 3; *P < 0.05, **P < 0.01, ***P < 0.001. Scale bars: (A and C) 2.2 cm; (F) 1 mm; and (G and I) 200 μm.

To simulate traumatic urethral injury, a full-thickness segment of 2.2 cm in length was surgically excised from the urethra of male New Zealand white rabbits, approximately 1.5 cm from the urethral orifice, to establish an irreversible injury model (Fig. 3A). Subsequently, a pre-constructed TEUG was implanted to bridge the defect. As controls, an autologous urethral graft and a cell-free PLA/gelatin nanofiber scaffold were also implanted to evaluate the regenerative performance of the TEUG. All 3 groups exhibited favorable surgical handling and suturability.

Postoperative observation revealed that indwelling catheters were naturally expelled 12 days after surgery. By day 60, rabbits in both the autologous graft and TEUG groups urinated freely, whereas those in the pure scaffold group developed urinary retention. Urethral pressure measurement using the BL420 biological signal acquisition system (Fig. 3B), combined with retrograde cystourethrography (Fig. 3C), confirmed that the neo-tissues formed in the autologous and TEUG groups maintained luminal patency and effectively transported contrast medium. In contrast, the pure scaffold group exhibited severe stenosis at the implantation site, with a lumen diameter of only 1.8 mm (Fig. 3D), necessitating urethrotomy to restore urinary function [25].

Physiological function testing revealed that both the autologous graft and TEUG responded to noradrenaline stimulation with smooth muscle contraction. However, the contraction rate in the TEUG group was slower than that in the autologous group, while the contraction amplitude in the pure scaffold group was significantly reduced (Fig. 3E). Macroscopic observation at 60 days post-implantation showed that the TEUG effectively preserved luminal openness and structural integrity, whereas the central region of the pure scaffold group exhibited mucosal obstruction and stenosis (Fig. 3F). Notably, the autologous graft group could not be visualized in cross-section due to its closed luminal state.

Histological analysis using MTC, SR, VVG, and AB staining revealed that the autologous graft group exhibited a star-shaped closed lumen structure in the absence of urine filling, with smooth muscle contraction inducing urethral wall folding (Fig. 3G and Fig. S6). In contrast, the TEUG group was in the stage of in vivo integration and remodeling. The newly formed smooth muscle layer began to contract, although incomplete scaffold degradation and immature tissue development resulted in mild wall folding and synchronized micro-fold structures on the luminal surface and within the ECM (Fig. 3G and H). In the pure scaffold group, insufficient tissue regeneration and ECM deposition led to disrupted luminal continuity (Fig. 3G and H).

Moreover, histological evaluation confirmed vascular ingrowth into both the TEUG and autologous graft scaffolds, with formation of a dense vascular network within the neo-tissue (Fig. 3G and H). No such vascularization was observed in the pure scaffold group. HE staining further revealed chronic inflammatory foci surrounding the neo-tissue in the pure scaffold group, characterized by disordered plasma cell infiltration and fibrous tissue hyperplasia (Fig. 3I). This may result from prolonged urine retention and local infiltration, leading to persistent inflammation. No such inflammatory changes were observed in the TEUG or autologous graft groups. Collectively, these findings demonstrate that the bioactive ECM interface within TEUGs facilitates mechanical integration of the neo-tissue, supports functional load transfer, and promotes physiological tissue remodeling.

### Bioactive ECM interface promotes structured growth of SMCs with enhanced mechanical load transmission

**Fig. 4.**
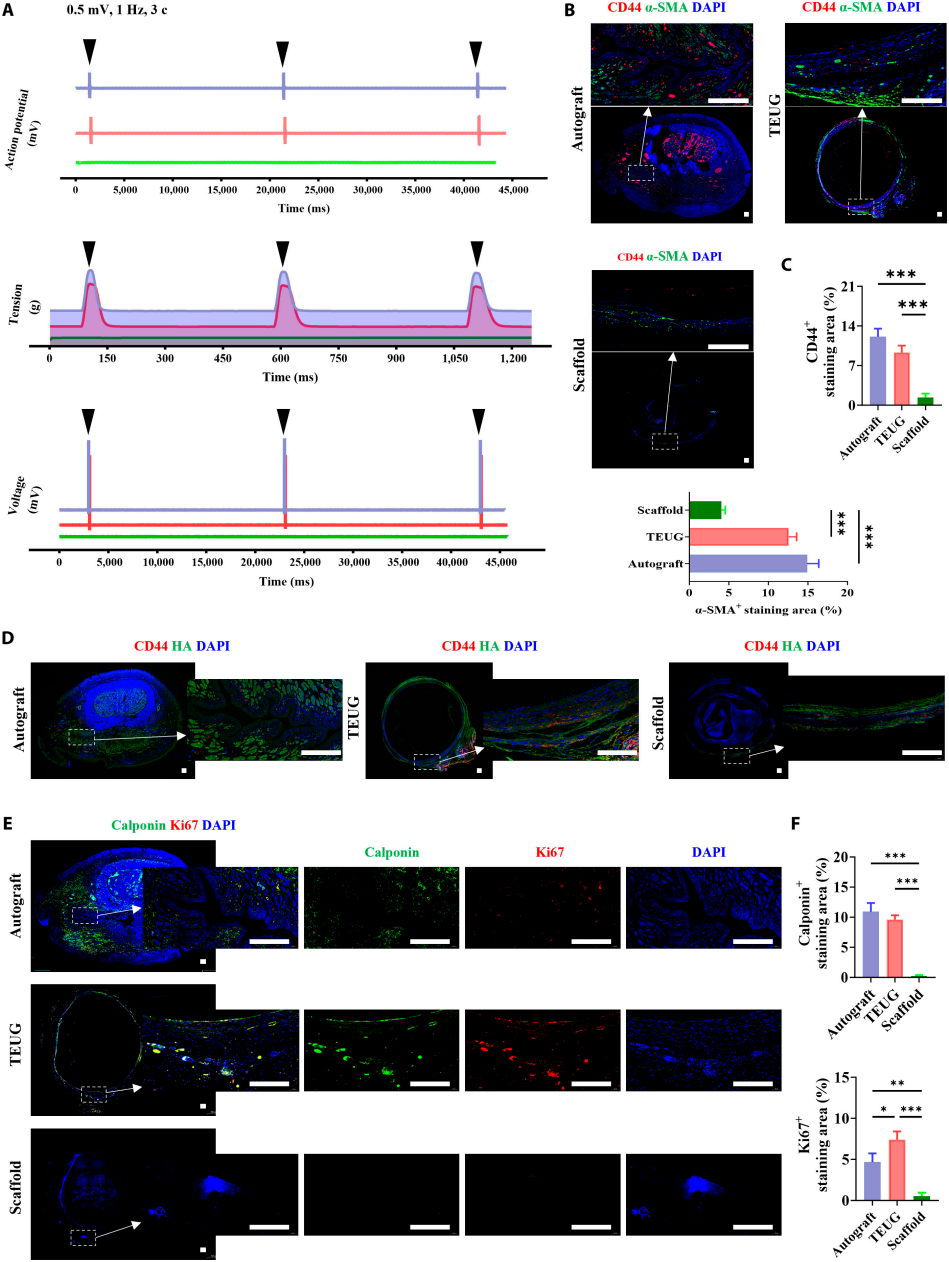
The bioactive ECM interface of TEUG can promote the structured growth of SMCs and enhance mechanical load conduction. By day 60 post-surgery, (A) representative action potential (top), tension (middle), and voltage (bottom) responses under 0.5 mV, 1 Hz stimulation (3 repeats). (B, D, and E) Immunostaining for CD44, α-SMA, HA, Calponin, and Ki67 in mid-cross-sections of retrieved urethral tissues. (C and F) Quantification of the percentage of CD44^+^ stem cells, α-SMA^+^ and Calponin^+^ SMCs, and Ki67^+^ proliferating cells. Data are mean ± SD; *n* = 3; *P < 0.05, **P < 0.01, ***P < 0.001. Scale bars: (B, D, and E) 250 μm.

To investigate the structural and functional integration of SMCs within the TEUG, we performed ex vivo electrophysiological assessments on grafts retrieved 60 days post-implantation, avoiding potential interference from in vivo physiological movements such as respiration and heartbeat. Under electrical stimulation conditions (0.5 mV, 1.0 Hz, 3 consecutive stimulations), both autografts and TEUGs exhibited clear action potential changes, indicating that the SMCs in these groups maintained normal electrical excitability, intact membrane ion channel function, and effective excitation–contraction coupling (Fig. 4A). In contrast, the scaffold-only group showed minimal response to stimulation, suggesting impaired cellular functionality (Fig. 4A).

Further analysis of mechanical responses revealed robust tension generation in the autograft group after each electrical pulse, reflecting strong intercellular signaling, contractile capability, and efficient electro-mechanical transduction (Fig. 4A). The TEUG group also demonstrated significant tension increases in response to stimulation, indicating well-coordinated SMC activity and preserved directional contractile function, albeit slightly lower than that of the autograft group. In contrast, the scaffold-only group failed to generate meaningful mechanical output, showing poor cellular coordination and limited contractile performance. Voltage curves confirmed consistent fluctuations in both the autograft and TEUG groups under stimulation, aligning with the observed patterns of action potentials and tension changes. These findings further support the presence of functionally mature SMCs capable of coordinated and directional contraction in these 2 groups, whereas the scaffold-only group displayed minimal voltage variation, highlighting its compromised responsiveness to external stimuli (Fig. 4A).

Immunofluorescence staining using classical SMC markers (α-SMA and calmodulin) revealed abundant mature SMC infiltration into the TEUG structure, with area percentages comparable to those in the autograft group (Fig. 4B and D and Figs. S7 and S8). Because of the folded architecture of native smooth muscle tissue in the autograft, positive cells were distributed in clusters, while in the TEUG, they exhibited a more evenly distributed pattern, indicative of structured growth guided by the bioactive ECM interface (Fig. 4C and F). Moreover, CD44-positive cell distribution in the TEUG was similar to that in the autograft (Fig. 4B and C), suggesting successful simulation of a native urethral microenvironment during TEUG construction. As a key stem cell surface receptor that binds to HA in the ECM [26], CD44 was found to specifically localize to HA-rich regions within the TEUG scaffold (Fig. 4D and Fig. S9), providing anchoring sites for endogenous stem cells and facilitating their sustained participation in tissue remodeling. A similar localization pattern was observed in the autograft (Fig. 4D and Fig. S10).

Cell proliferation assays further demonstrated regular Ki67-positive cell distribution along the luminal and wall layers of the TEUG. Given that the autograft only experienced partial cell loss at trauma and suture sites, while both the TEUG and scaffold groups underwent full-thickness urethral reconstruction, the number of proliferative cells in the TEUG group was significantly higher than that in the autograft group (Fig. 4E and F). This suggests that the structural and biochemical support provided by the TEUG created a favorable microenvironment for organized cell proliferation, thereby maintaining urinary transport function [6,25,27,28]. In contrast, the scaffold-only group lacked functional seed cells and active signaling cues, resulting in unstable attachment of endogenous stem cells (Fig. 4B to D and Figs. S11 and S12), fewer Ki67-positive cells, and poor development of α-SMA-, calponin-, and CD44-positive populations (Fig. 4B to F). Taken together, these results demonstrate that the bioactive ECM interface within TEUGs effectively promotes the structured growth of SMCs, supports mature electrophysiological function, and enhances mechanical load transmission.

### ECM-mediated immune modulation by GAGs facilitates pro-repair macrophage infiltration post-catheter detachment

**Fig. 5.**
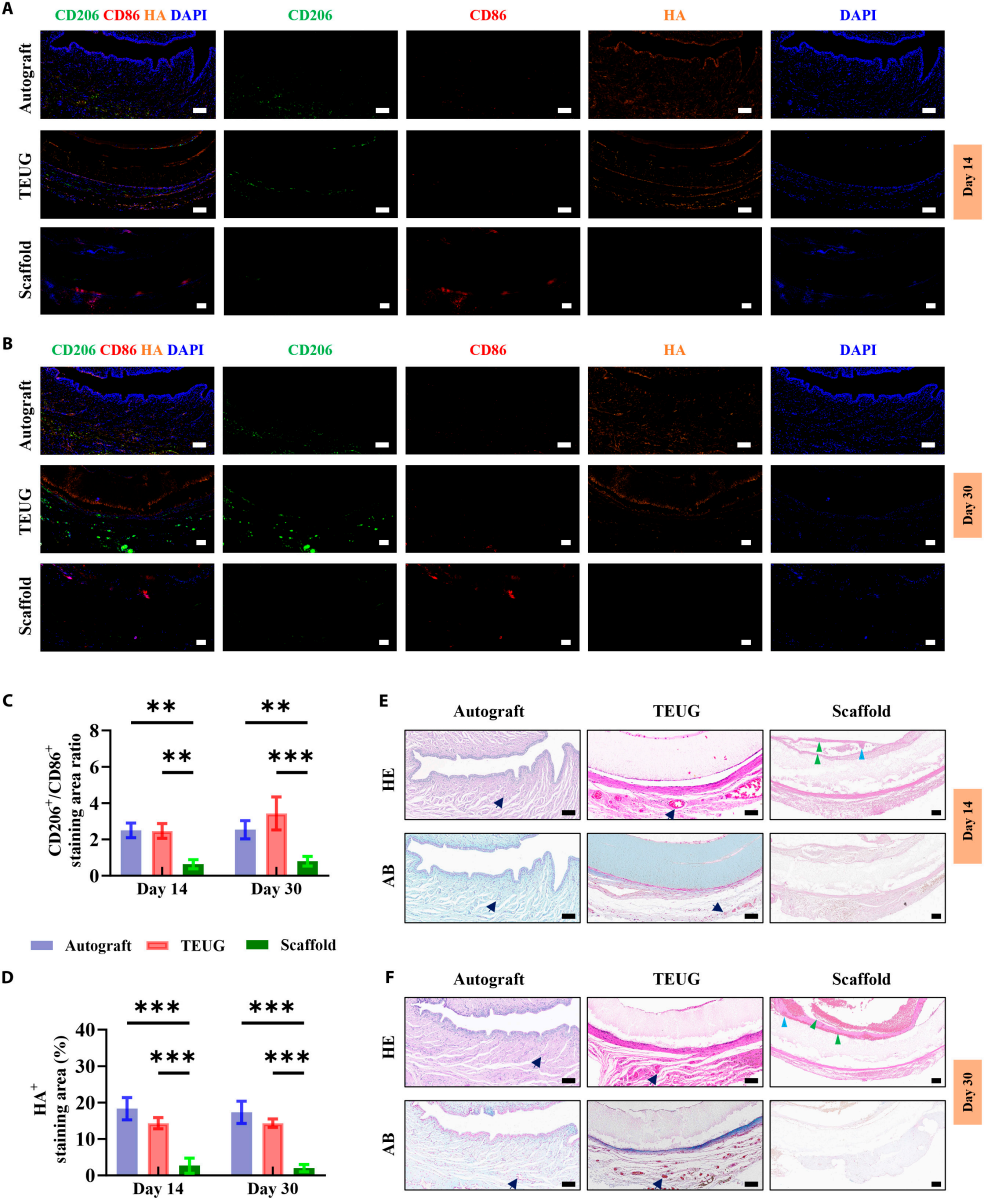
GAGs in the ECM interface of TEUG regulate the immune microenvironment at the defect site. (A and B) Immunostaining for CD206, CD86, and HA in mid-cross-sections of retrieved urethral tissues at 14 (A) and 30 (B) days post-implantation. (C and D) Quantification of the CD206^+^/CD86^+^ macrophage ratio and HA levels in tissue sections. Data are mean ± SD; *n* = 3; **P < 0.01, ***P < 0.001. (E and F) HE and AB staining of mid-cross-sections from retrieved urethral tissues at 14 (E) and 30 (F) days post-implantation. Black arrow: microvessel; green triangle: lymphocyte infiltration; blue triangle: local tissue edema. Scale bars: (A, B, E, and F) 100 μm.

To elucidate how TEUG interacts with the host immune system during early tissue remodeling following spontaneous catheter detachment, we collected mid-bridge tissue samples at 14 and 30 days post-implantation. Immunofluorescence staining revealed that immune cells were predominantly clustered around the graft interface. Notably, both autografts and TEUGs exhibited a dense deposition of HA-rich ECM, which was closely associated with the infiltration of CD206^+^ pro-repair macrophages, with minimal presence of CD86^+^ pro-inflammatory macrophages (Fig. 5A and B). In contrast, the scaffold-only group lacked functional ECM components and was primarily infiltrated by CD86^+^ macrophages, with significantly fewer CD206^+^ cells (Fig. 5A and B). Quantitative analysis showed that the CD206^+^/CD86^+^ staining area ratio in the autograft group remained consistently high throughout the remodeling phase, reaching (2.5 ± 0.4) at day 14 and (2.5 ± 0.6) at day 30. The TEUG group displayed similar or even higher ratios, increasing from (2.4 ± 0.4) at day 14 to (3.4 ± 1.2) at day 30, indicating progressive recruitment of M2-like macrophages as tissue repair advanced. In comparison, the scaffold group exhibited significantly lower ratios of (0.6 ± 0.2) and (0.8 ± 0.2) at the same time points, reflecting a persistent inflammatory response dominated by M1-type macrophages (Fig. 5C).

Further correlation analysis revealed that the HA^+^ staining area across all groups aligned closely with the CD206^+^/CD86^+^ ratio (Fig. 5D), reinforcing the role of GAG-rich ECM in promoting an anti-inflammatory and regenerative immune microenvironment. Histological examination using HE staining confirmed the absence of chronic inflammation or fibrosis in the autograft and TEUG groups, whereas the scaffold group exhibited plasma cell infiltration and fibrotic changes, consistent with ongoing inflammatory responses (Fig. 5E and F). Importantly, despite the presence of some inflammatory cells, the TEUG group demonstrated robust neovascularization surrounding the graft, likely contributing to the efficient clearance of metabolic waste and inflammatory mediators. These newly formed vessels not only supported nutrient delivery but also facilitated the rapid influx of circulating monocytes and lymphocytes, which aided in debris clearance and further dampened local inflammation. In contrast, limited vascular ingrowth in the scaffold group may have hindered immune resolution and contributed to the sustained M1 polarization (Fig. 5E and F). AB staining for acidic GAGs showed abundant deposition in both autograft and TEUG tissues at 14 and 30 days post-surgery (Fig. 5E and F), corroborating the immunofluorescence findings and underscoring the role of GAGs in shaping a permissive immune environment for tissue regeneration. Moreover, AB staining revealed significant microvascular development in TEUG and autograft tissues, while the scaffold group exhibited poor vascularization—a trend consistent with observations made at the later 60-day time point (Fig. 2G to I). Together, these results demonstrate that the GAG-enriched ECM interface within TEUG actively modulates the host immune response after catheter detachment, facilitating the selective infiltration of CD206^+^ macrophages and suppressing pro-inflammatory M1 polarization.

### The bioactive interface of TEUG orchestrates innate and adaptive immune cell recruitment to establish a regenerative microenvironment

**Fig. 6.**
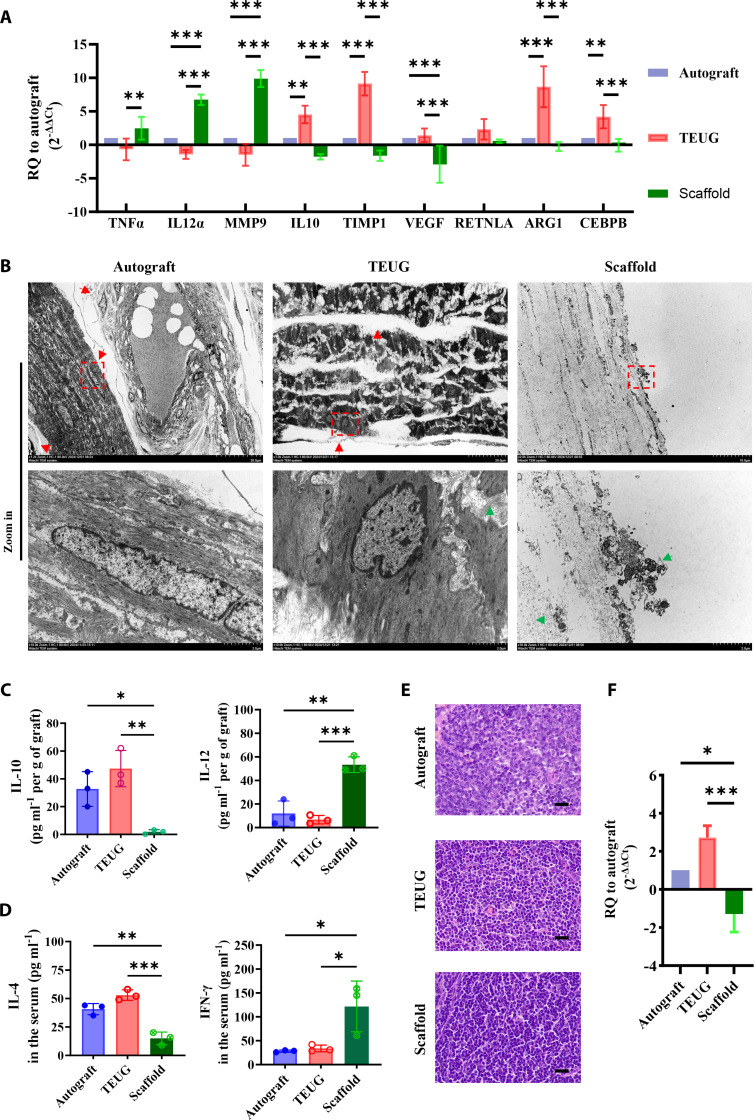
The bioactive interface of TEUG constructs a regenerative immune microenvironment by coordinating the recruitment of innate and adaptive immune cells. (A) qRT-PCR analysis of gene expression in flow-sorted monocytes from autograft, TEUG, and pure scaffold samples 30 days post-implantation. Data are relative quantitation (RQ) normalized to autograft (RQ = 2^−∆∆Ct^), shown as mean ± SD (*n* = 3; **P < 0.01, ***P < 0.001). (B) TEM images of the urethral wall in the central neo-tissue region of each graft type. Red arrow: GAGs in the urethral ECM; green arrow: nanofibers in TEUG and scaffold groups. (C) Secretion levels of IL-10 and IL-12 in graft samples 30 days post-implantation. Data are mean ± SD; *n* = 3; *P < 0.05, **P < 0.01, ***P < 0.001. (D) Systemic levels of IL-4 and IFN-γ in treated animals 30 days post-implantation. Data are mean ± SD; *n* = 3; *P < 0.05, **P < 0.01, ***P < 0.001. (E) HE staining of inguinal lymph nodes after 30 days of treatment with autograft, TEUG, or scaffolds (scale bar: 20 μm). (F) qRT-PCR analysis of *Il4* gene expression in inguinal lymph nodes 30 days post-treatment (RQ = 2^−∆∆Ct^; mean ± SD, *n* = 3; *P < 0.05, ***P < 0.001).

To elucidate how the bioactive interface of TEUG shapes the immune microenvironment during early urethral remodeling, we analyzed macrophage polarization states and systemic immune responses by collecting tissue samples from the upstream anastomotic region at 30 days post-implantation. Transcriptomic analysis of isolated macrophages via qRT-PCR revealed that TEUG induced a gene expression profile dominated by the M2 phenotype, characterized by up-regulated expression of *ARG1* [29], *CEBPB*, and *TIMP-1*, along with down-regulation of pro-inflammatory markers such as *MMP9* and *IL-12a* (Fig. 6A). Notably, VEGF expression was also elevated in the TEUG group, suggesting enhanced angiogenic and immunomodulatory activity, which may contribute to improved tissue repair and anti-fibrotic effects. In contrast, the scaffold-only group exhibited a classical M1-like polarization pattern, marked by increased expression of *MMP9* and *IL-12a*, and reduced levels of *VEGF*, *TIMP-1*, and *IL-10*, indicating a persistent inflammatory environment unfavorable for regeneration (Fig. 6A).

Ultrastructural analysis using TEM further supported these findings. In the autograft group, a well-organized multilayered structure had formed by day 30, including mature urothelial cells with clear cell boundaries and intact basement membranes. Collagen and elastic fibers were arranged in a reticular pattern, interspersed with GAG-rich regions exhibiting low electron density (Fig. 6B). In TEUG-treated tissues, the luminal surface was covered by a uniform, amorphous mucosal-like layer consistent with GAG deposition, beneath which host cells showed regular morphology and membrane structures. These observations align with histological findings of GAG accumulation and neo-tissue formation. In contrast, the scaffold-only group lacked cellular infiltration or mucosal coverage, reflecting poor integration with host tissue (Fig. 5F).

Local cytokine profiling via ELISA confirmed that both autograft and TEUG groups exhibited elevated levels of the anti-inflammatory cytokine IL-10 and reduced secretion of the pro-inflammatory mediator IL-12 (Fig. 6C), indicative of a tolerogenic local immune microenvironment. Systemic immune analysis further revealed that plasma levels of IL-4 were significantly increased, while IFN-γ levels were decreased in TEUG recipients (Fig. 6D), suggesting activation of a Th2-type adaptive immune response. In contrast, the scaffold group displayed the opposite trend—elevated IFN-γ and reduced IL-4—consistent with a dominant Th1-type inflammation. To further verify whether the autograft and TEUG can induce a systemic Th2-like adaptive immune response, we collected the inguinal draining lymph nodes of the animals in each group for HE staining analysis [30]. Histopathological evaluation of draining lymph nodes revealed lymph node hypertrophy in both TEUG and scaffold groups compared to autografts (Fig. 6E). However, only the TEUG group showed significantly elevated IL-4 expression in lymph nodes (Fig. 6F), confirming its capacity to induce a coordinated innate-adaptive immune response favoring tissue regeneration. Taken together, these findings demonstrate that the bioactive interface of TEUG actively orchestrates both innate and adaptive immune components—promoting M2 macrophage polarization and inducing a Th2-skewed systemic immune response—thereby establishing a regenerative immune microenvironment essential for successful urethral reconstruction.

### Stem cell homing to the TEUG interface contributes to functional tissue repair

Understanding which cell populations contribute to neo-tissue formation at the defect site and assume load-bearing functions after internal catheter removal is critical for evaluating the regenerative capacity of engineered grafts [16,18,31]. Given that bioactive scaffolds can modulate the host immune microenvironment and recruit endogenous progenitor cells, we sought to determine whether TEUG could guide stem cell homing to support functional urethral regeneration.

**Fig. 7.**
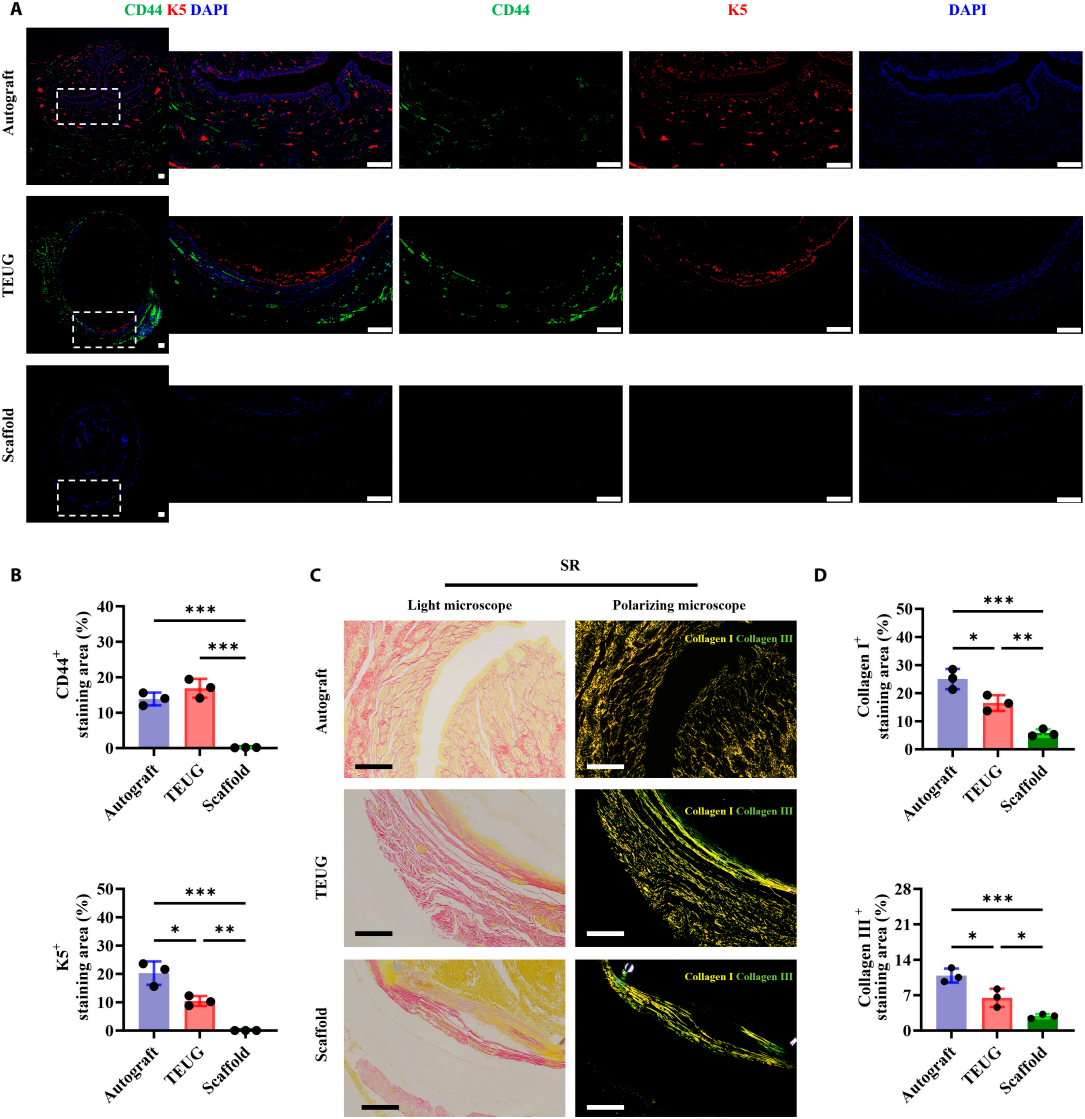
TEUG interface facilitates homing of endogenous progenitor stem cells. (A) Immunostaining for CD44 and K5 in mid-cross-sections of retrieved urethral tissues at 30 days post-implantation. (B) Quantification of CD44^+^ stem cell and K5^+^ progenitor cell percentages. Data: mean ± SD; *n* = 3; *P < 0.05, **P < 0.01, ***P < 0.001. (C) SR staining of mid-cross-sections from retrieved urethral tissues at 30 days post-implantation (light microscopy, left; polarized light, right). Scale bar: 200 μm. (D) Quantification of collagen I and III staining percentages in different grafts.

To address this, we analyzed the spatial distribution of endogenous progenitor and stem cells within the urethral wall 30 days post-implantation (18 days after catheter removal). Immunofluorescence staining for CD44 (a general stem cell marker) and K5 (a urethral progenitor cell marker) revealed distinct patterns across groups. In autograft-treated urethras, CD44^+^ cells were predominantly localized in pericorpuscular adipose tissue, connective tissue, bone marrow, and urethral glands, while K5^+^ cells were mainly found in the basal and suprabasal layers of the epithelium and within urethral glands (Fig. 7A and Fig. S13). These findings suggest that autografts effectively recruit endogenous stem and progenitor cells to the injury site, where they differentiate into mature urothelial or glandular cells, contributing to tissue repair.

In TEUG-treated animals, CD44^+^ stem cells were primarily localized to the outer wall and interstitial spaces of the lumen, whereas K5^+^ progenitor cells accumulated along the luminal inner wall and within urethral glands (Fig. 7A and Fig. S14), indicating active recruitment and directed migration toward the bioactive interface. In contrast, almost no CD44^+^ stem cell or K5^+^ progenitor cell signals were detected in the scaffold group (Fig. 7A and Fig. S15). Quantitative analysis confirmed that the proportion of CD44^+^ cells in the TEUG group was comparable to that in the autograft group and significantly higher than that in the scaffold-only group (Fig. 7B). Although the percentage of K5^+^ cells was lower in TEUG compared to autografts (*P* < 0.05), it remained significantly elevated relative to the scaffold group (*P* < 0.01).

Importantly, SR staining and polarized light microscopy revealed that collagen I and III fibers in TEUG tissues formed an interwoven structural network centered on the graft interface, resembling native urethral architecture. Similarly, autograft tissues exhibited a cross-linked collagen matrix typical of healthy urethral tissue (Fig. 7C and D). In contrast, the scaffold group showed only layered collagen deposition forming an immune-isolation barrier, characterized by inflammatory infiltration and vascular occlusion (Fig. 7C and D), suggesting poor integration and limited regenerative potential. These results demonstrate that the bioactive interface of TEUG actively supports stem cell homing, guiding endogenous progenitors to integrate into the remodeling tissue and participate in functional tissue repair. The structured ECM and immunomodulatory properties of TEUG appear to create a permissive niche that promotes stem cell recruitment and differentiation, ultimately enabling successful urethral regeneration.

## Discussion

The ideal urethral reconstruction surgery aims to restore the normal physiological functions of the urinary system and minimize the risk of postoperative complications [6]. In recent years, significant progress has been made in improving the therapeutic efficacy of TEUGs, particularly in the field of immunomodulation [7,9,16,18,31,32]. However, FBR and acute inflammation induced by implantation remain critical factors affecting the integration and repair efficacy of TEUGs. To address these issues, we propose a novel TEUG construction strategy based on building a bionic tissue interface. By co-culturing rabbit-derived SMCs and ECs with nanofiber scaffolds, “autologous cellularized” TEUGs were constructed in vitro. These cells secreted and deposited ECM on the scaffolds, forming a bioactive interface with anti-FBR capabilities. This interface not only has the ability to inhibit FBR at the structural and biochemical levels but also effectively alleviates acute inflammatory responses and promotes vascular network reconstruction, orderly proliferation of new tissues, replacement of original ECM, establishment of mechanical properties of new tissues, and maturation of functional structures.

Compared with previous strategies, such as hydrogel coatings [15,16], bionic patches [17,18], decellularized tissue matrices (DTMs) [33,34], growth factor (GF) delivery systems [35,36], and smartly designed surface-modified scaffolds [16,18,31], the bioactive interface constructed in this study offers superior stability, immunoregulatory efficiency, and functional integration. DTMs retain native ECM composition and architecture [33], but often contain residual immunogenic components (e.g., cell debris or xenogeneic proteins) that provoke excessive FBR [34], and suffer from poor mechanical integrity and rapid in vivo degradation, leading to graft contracture or collapse [1]. In contrast, our interface is built from autologous cells in vitro, eliminating immunogenic risk, while the nanofibrous scaffold provides urethra-matched mechanical support to prevent structural failure during regeneration.

Conventional GF delivery systems (e.g., heparin-functionalized scaffolds loaded with TGF-β or VEGF) are limited by rapid GF degradation in vivo [35,36], necessitating high doses or complex release systems that may cause side effects like tissue hyperplasia [37]. Our approach leverages co-cultured cells to secrete an endogenous ECM enriched with spatiotemporally coordinated bioactive factors—including basic fibroblast growth factor and platelet-derived growth factor [6,7], enabling physiological immune modulation without exogenous GFs, thereby enhancing both safety and regenerative synchrony.

Recent advances highlight the importance of engineering intrinsic scaffold properties to guide immune responses. Examples include the following: a sodium alginate/gelatin/reduced graphene oxide patch that directs macrophage polarization via stiffness and topography for scarless healing [18]; hypoxia-mimicking heterogeneous scaffolds that promote angiogenesis while dampening inflammation through endogenous pathways [31]; and multilayered hydrogels with urethra-matched elasticity that sustain M2 macrophage dominance and reduce fibrosis without exogenous cytokines [16].

Building on these concepts, our bioactive interface functions as a dynamic “living” construct: seeded SMCs and ECs continuously secrete and remodel ECM during culture and after implantation, maintaining long-term immunomodulation [14,26]. For instance, cell-replenished HA supports persistent M2 polarization and suppresses pro-inflammatory responses throughout regeneration [19,20].

Unlike single-function approaches targeting FBR inhibition or mechanical support [15,17], our system integrates multiple regenerative cues: anisotropic wettability and elasticity minimize immune recognition [38]; ECM components (e.g., GAGs) modulate macrophage and T-cell responses [30,39]; and the scaffold architecture guides endogenous stem cell proliferation and differentiation [20,40]. This multidimensional, self-sustaining mechanism simultaneously mitigates FBR and drives functional urethral regeneration—surpassing the limitations of single-target strategies.

Compared with previous strategies, such as hydrogel coatings [15,16] or bionic patches [17,18], the interface constructed in this study has advantages in terms of stability and immunoregulatory ability [6,7,9,10,16,22]. The tissue interface has anisotropic wettability and elasticity similar to natural urethral tissue, which can effectively reduce immune recognition, consistent with findings that fiber flexibility reconciles matrix recruitment and fiber modulus to promote cell mechanosensing [38]. Its ECM is rich in neutral and acidic polysaccharide components, among which HA is particularly critical, as it can not only inhibit the polarization of pro-inflammatory M1-type macrophages but also promote the differentiation of regenerative M2-type macrophages [4,15,16,18]. These M2-type macrophages further release cytokines beneficial for tissue regeneration, such as IL-10 and IL-4, thereby reshaping the host immune microenvironment after urethral injury. Notably, elevated IL-4 levels can trigger the activation of Th2 cells in adaptive T lymphocytes, thereby enhancing local regenerative immune responses [30,39,41]. This immunoregulatory cascade is crucial for guiding the differentiation of endogenous stem cells toward functional SMCs, thereby significantly enhancing tissue repair.

In addition to immunoregulatory effects, the bioactive ECM interface supports dynamic cellular changes during regeneration. The urethra and its surrounding areas (such as blood vessels [42,43], nerves [44], and skin [45]) are rich in various endogenous progenitor stem cells, which can synergistically participate in tissue repair after injury [20]. The interface we constructed not only provides temporary structural frameworks and adhesion sites for these cells but also guides their orderly proliferation and differentiation through biochemical signals. In particular, GAG-type ECM components enhance the homing and integration ability of stem cells and promote the recovery of urethral load-bearing and conduction functions.

Furthermore, collagen and elastin play the roles of “structural scaffold” and “elastic core”, respectively, in the process of urethral regeneration. Through the regulation of mechanical synergy, time matching, and cell signaling pathways, they jointly determine the quality of functional reconstruction of new tissues, which aligns with the concept that fibrous pattern orientation and modulus synergistically influence cellular mechanoresponse [46]. These findings are consistent with previous research results [16,18–20,31], further verifying the scientific hypothesis that regulating the biophysical and biochemical properties of biomaterials can effectively guide the remodeling of the extracellular microenvironment, thereby recruiting endogenous cells to participate in urethral tissue regeneration. Similar to the major advances made in cancer research through T-cell immunotherapy, the concept of “immune-guided regeneration” is gradually being introduced into the design of TEUGs, offering novel insights and therapeutic strategies for improving urethral repair and regeneration [47].

More importantly, this study is the first to systematically compare the morphological characteristics, histological manifestations (including ECM components such as type I collagen, type III collagen, elastin, and GAGs), and immunofluorescence staining results of vascular wall cross-sections between the in vitro constructed autologous urethra-like TEUGs and those 60 days after implantation. This comparison provides direct evidence for the first time that the seeded ECs and SMCs and their secreted ECM not only provide structural support for endogenous stem cells but also effectively guide their directional differentiation into mature SMCs, thereby reconstructing the load-bearing and conduction functions of the urethra. Not setting up a “cell-only” control group is fully scientifically justified: it is mainly for cell sheet research (requiring specialized equipment and long-term culture [48,49]), free cells fail to stably colonize the urethral defect (a transient urinary organ) or mimic our “cell–scaffold–matrix” microenvironment, and scaffold-free pure cell sheets form tube-like structures in only 20% of cases with weak mechanics insufficient for urethral repair [1]. Thus, omitting this group does not compromise the study’s scientific validity or reliability.

Despite the promising progress, this study has several limitations. The dominant endogenous stem or progenitor cell population driving regeneration, whether urothelial basal cells, periurethral mesenchymal stem cells, or gland-resident progenitors [40,50], remains unclear. Moreover, findings from the rabbit autologous model may not fully translate to humans due to differences in urethral anatomy, immune response, and healing kinetics; validation in large-animal models (e.g., porcine or canine) is therefore needed. Long-term follow-up beyond 60 days is also lacking, with critical questions about graft durability, stricture recurrence, and functional recovery (e.g., urinary flow and sexual function) unresolved. Although the 2-week in vitro culture window meets clinical feasibility for elective surgery, scaling up production under Good Manufacturing Practice standards while ensuring sterility, reproducibility, and quality control presents a significant translational challenge. Finally, deeper mechanistic insights, particularly the molecular crosstalk between the HA-rich bioactive interface, immune cells, and specific regenerative cell subsets, are required, ideally through single-cell or lineage-tracing approaches, to fully realize the potential of this “immune-guided regeneration” strategy for clinical urethral reconstruction.

In conclusion, to overcome the functional impairment caused by FBR at the interface between implanted materials and host tissues, this study developed a TEUG with anti-FBR and anti-inflammatory properties. By co-culturing rabbit-derived SMCs and ECs on nanofibrous scaffolds fabricated from clinically approved materials, we successfully constructed a bioactive interface with autologous-like characteristics. This approach achieves cell-seeded TEUG fabrication in 2 weeks, fitting within the clinical timeline of elective urethroplasty. Furthermore, the engineered bioactive ECM interface significantly improved TEUG patency, urine transport capacity, synchronized contractility, and directional action potential conduction, demonstrating superior functional integration. Our findings provide a promising strategy for enhancing the in vivo integration and long-term functionality of TEUGs, offering a solid experimental foundation for future clinical translation.

## Data Availability

All data generated during the study are presented in the manuscript and the Supplementary Materials.
