# Supplementary material for: Construction of a Bioactive ECM Interface Enables Concurrent Suppression of Foreign Body Reaction, Inflammation, and Promotion of Urethral Regeneration
Source: Biomater Res. 2026 Mar 11;30:0334. doi: 10.34133/bmr.0334 (PMC12976687; doi:10.34133/bmr.0334)
Supplement: Supplementary 1 — Figs. S1 to S15 Table S1 [file bmr.0334.f1.docx]

*Supporting Information*

# Construction of a Bioactive ECM Interface Enables Concurrent Suppression of Foreign Body Reaction, Inflammation, and Promotion of Urethral Regeneration

Peihong Han ^1,#,^ Xinyu Lei^1,#^, Shutong Li^1,#^, Kai Fu^2^, Xiuhong Sun^2^, Rui Zhou^2^, Yuqing Niu ^1,*^

1. School of Life and Health Technology, Dongguan University of Technology, Dongguan, 523808, China
2. Provincial Key Laboratory of Research in Structure Birth Defect Disease and Department of Pediatric Surgery, Guangzhou Women and Children’s Medical Center, Guangzhou Medical University, Guangzhou, 510623, Guangdong, China

Correspondence: [niuyuqing@dgut.edu.cn](mailto:niuyuqing@dgut.edu.cn) [yuqing944@126.com](mailto:yuqing944@126.com) (Y. Niu)

^#^ These authors contributed equally to this work.

# S1. Experimental section

# S1.1 Cell isolation and culture

After general anesthesia of the animal, a small incision was made above the pubic symphysis to expose the bladder. A tissue sample measuring 1.0 cm × 0.5 cm × 0.7 cm was obtained from the top of the bladder wall. After rinsing with 2% penicillin-streptomycin (TMS-AB2, Sigma-Aldrich) phosphate buffered saline (PBS, 10010023, Gibco^TM^) solution, the tissue was transferred to 2 mL of a dissociation solution composed of filtered Hank's Balanced Salt Solution (HBSS, H4891, Sigma-Aldrich) (without Ca²⁺ and Mg²⁺), 10 mM 4-(2-hydroxyethyl)-1-piperazineethanesulfonic acid (HEPES, 215001000, Gibco^TM^), 1% penicillin-streptomycin, 0.1 mg/mL aprotinin (A3886, Sigma-Aldrich), and 2.4 U/mL Dispase II (17105041, Gibco^TM^), and stored overnight at 4°C.

The next day, fat, blood vessels, peritoneum, and epithelial mucosa were gently removed using microdissecting forceps. The remaining bladder smooth muscle tissue was minced into small pieces and digested with 1.5 U/mL Liberase (05401020001, Sigma-Aldrich) at 37°C for 90 min to obtain a tissue extract containing individual cells. The extract was further filtered through a 70 μm cell strainer (CLS431751, Corning®). The filtrate was collected in a 50 mL tube (332260, Nunc^TM^) and centrifuged at 187×g for 6 min. The supernatant was carefully discarded, and the precipitated cells were resuspended in 3 mL of Dulbecco’s modified Eagle’s medium (DMEM,11885084, Gibco^TM^) containing 10% fetal bovine serum (FBS, A5670801, Gibco^TM^), 1% smooth muscle cell growth supplement (S00725, Gibco^TM^), and 1% penicillin-streptomycin. Subsequently, the primary SMCs isolated from the bladder were transferred to a T-25 culture flask (CLS431463, Corning®) for further proliferation.

For the isolation of bladder ECs, the epithelial mucosa samples were rinsed three times with 0.25% chloramphenicol (C1919, Sigma-Aldrich) solution and PBS, minced into small pieces, and then digested with 2.5 U/mL Dispase II (17105041, Gibco^TM^) at 4°C for 15h to obtain a single-cell suspension of primary bladder ECs. Digestion was terminated by the addition of 10% FBS. The filtration and centrifugation methods for bladder ECs were the same as those for SMCs. The primary culture medium for bladder ECs was DMEM medium supplemented with 10% FBS.

Cells were cultured in a 95% humidified incubator at 37°C with 5% CO₂, and the medium was changed every other day. In subsequent experiments, we used SMCs and ECs from passages 3 to 4.

# S1.2 Water contact angle measurement

To measure the contact angles of the TEUGs created *in vitro* and the scaffolds, an optical contact angle meter (Theta Flex, Biolin Scientific, Finland) was utilized. Briefly, the samples were placed flat on the instrument's stage. A 5-μL deionized water droplet was dispensed onto the surface. High-frame-rate images were captured over 30-60 s. The software then analyzed the droplet profile to calculate the contact angle. Five measurements per sample were averaged to ensure accuracy.


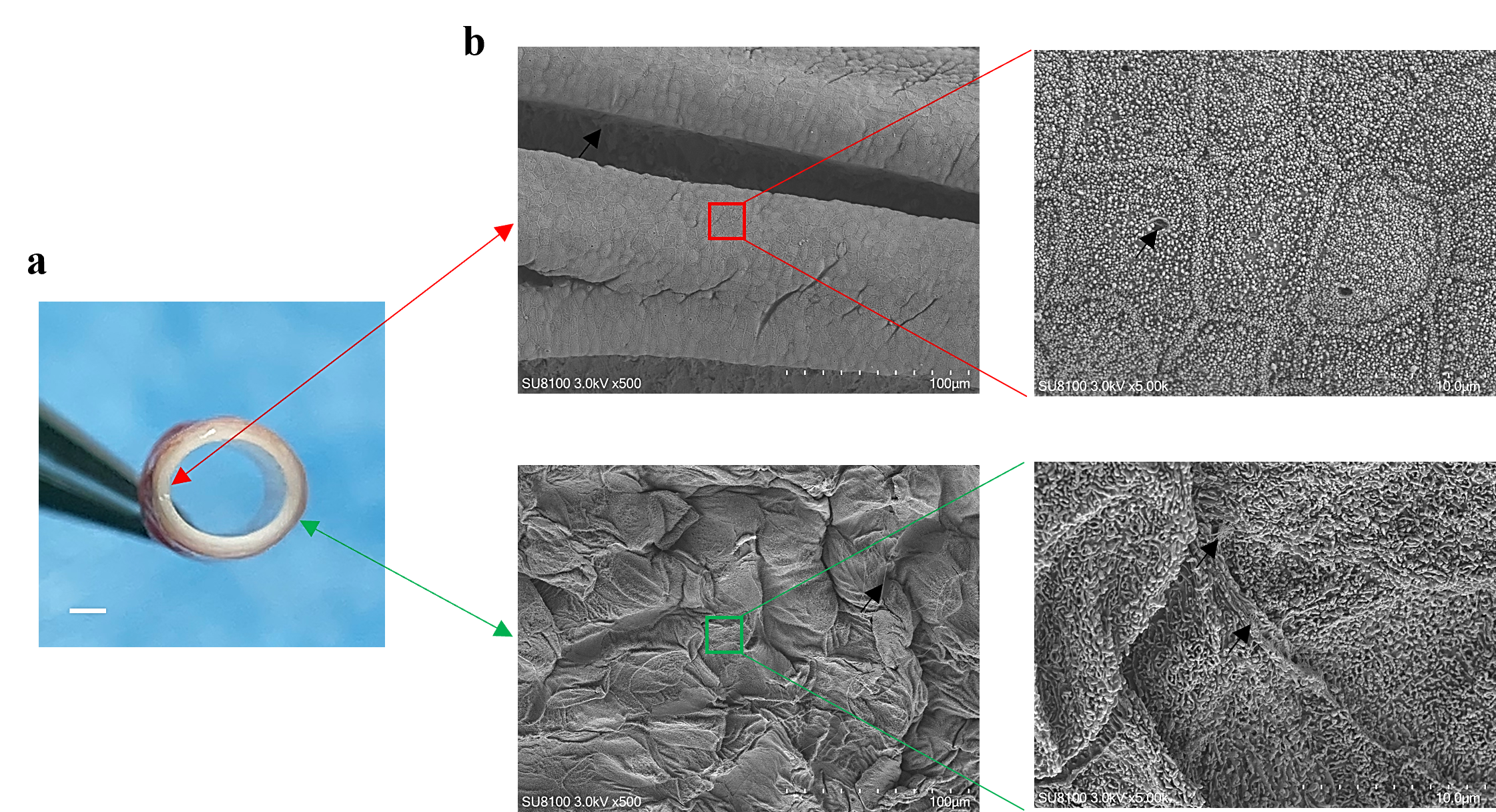


**Figure S1. Macroscopic and microscopic characteristics of TEUG after 7 days of *in vitro* co-culture with ECs and SMCs.** a. Digital image of the cross-sectional view of TEUG. Scale bar: 1 mm. b. SEM images showing the microscopic morphology of ECs and SMCs on the luminal and abluminal surfaces of TEUG. The black arrows indicate the scaffold nanofibers that have not yet been fully covered by ECs and SMCs.


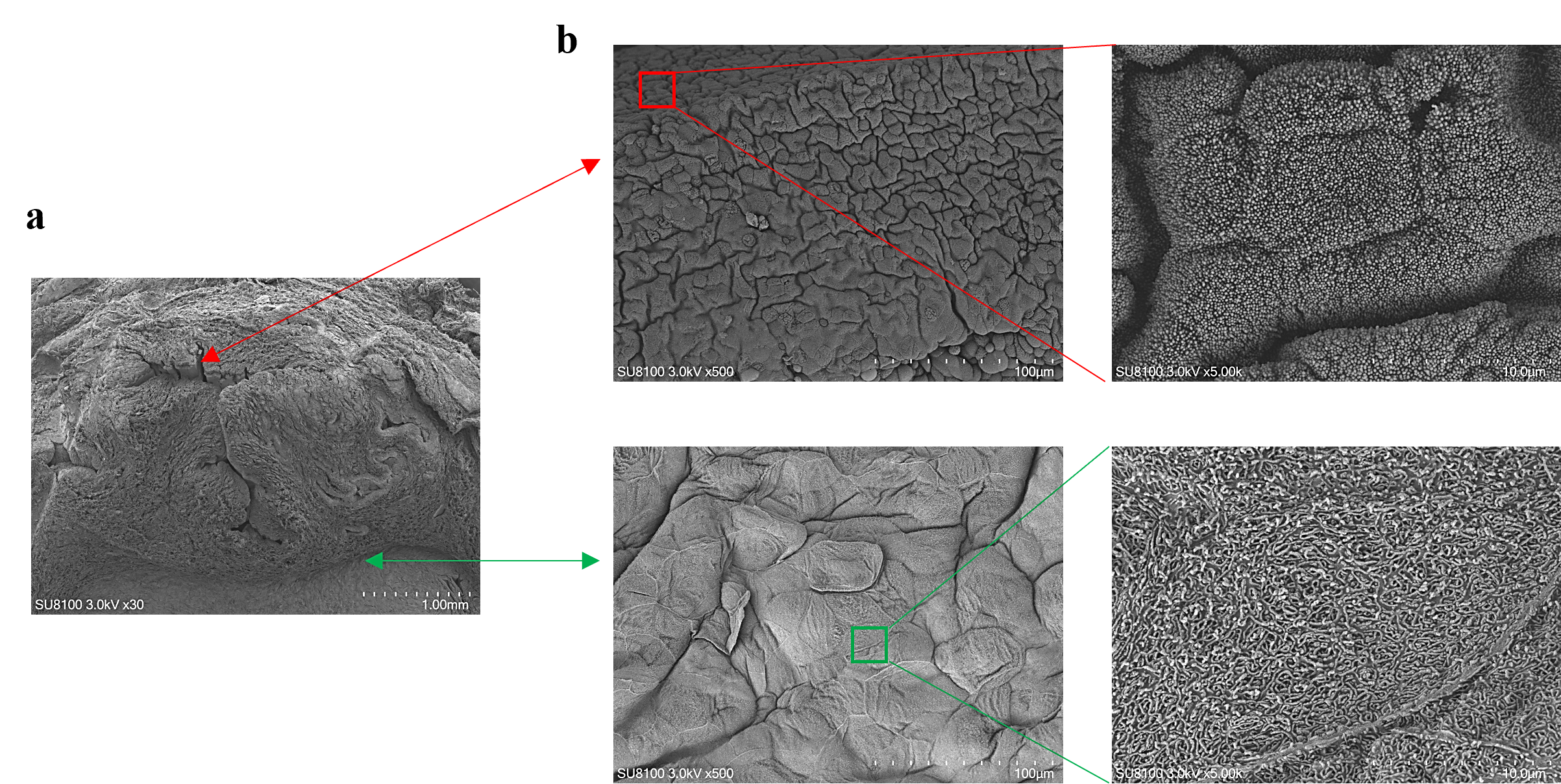


**Figure S2. Morphology of the cross-section and the inner and outer surfaces of the lumen of the urethra in juvenile rabbit.** a. SEM image of the cross-section of the urethra in juvenile rabbits at a low magnification. b. SEM images of the inner and outer surfaces of the lumen of the urethra in juvenile rabbits at a high magnification.


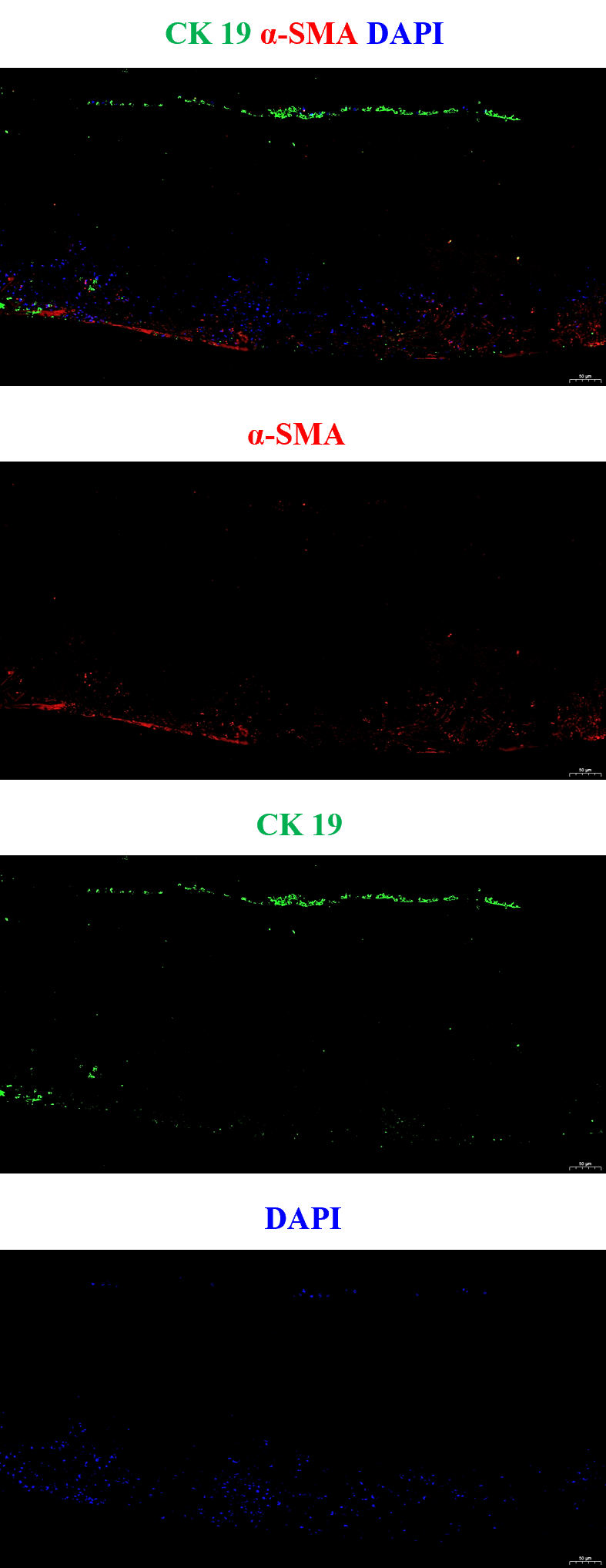


**Figure S3. Immunostaining of CK19 and α-SMA on cross-section of cell-laden TEUG tissue engineering constructs successfully constructed *in vitro*.** From top to bottom: merged channel, α-SMA channel, CK19 channel, DAPI channel.


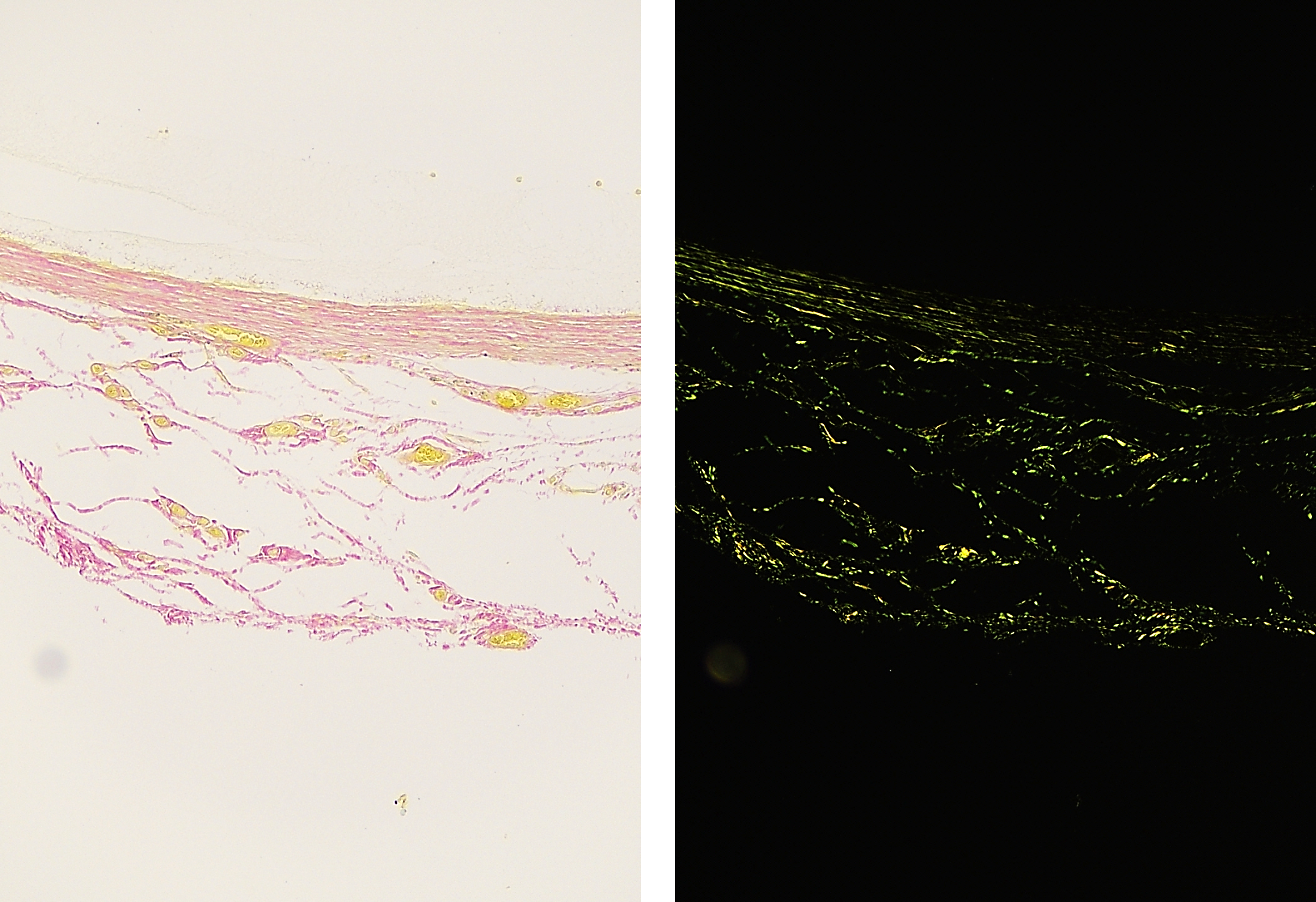


**Figure S4. SR staining of cross-section of cell-laden TEUG tissue engineering constructs successfully constructed *in vitro*. Left panel: light microscope; right panel: polarized microscope.**


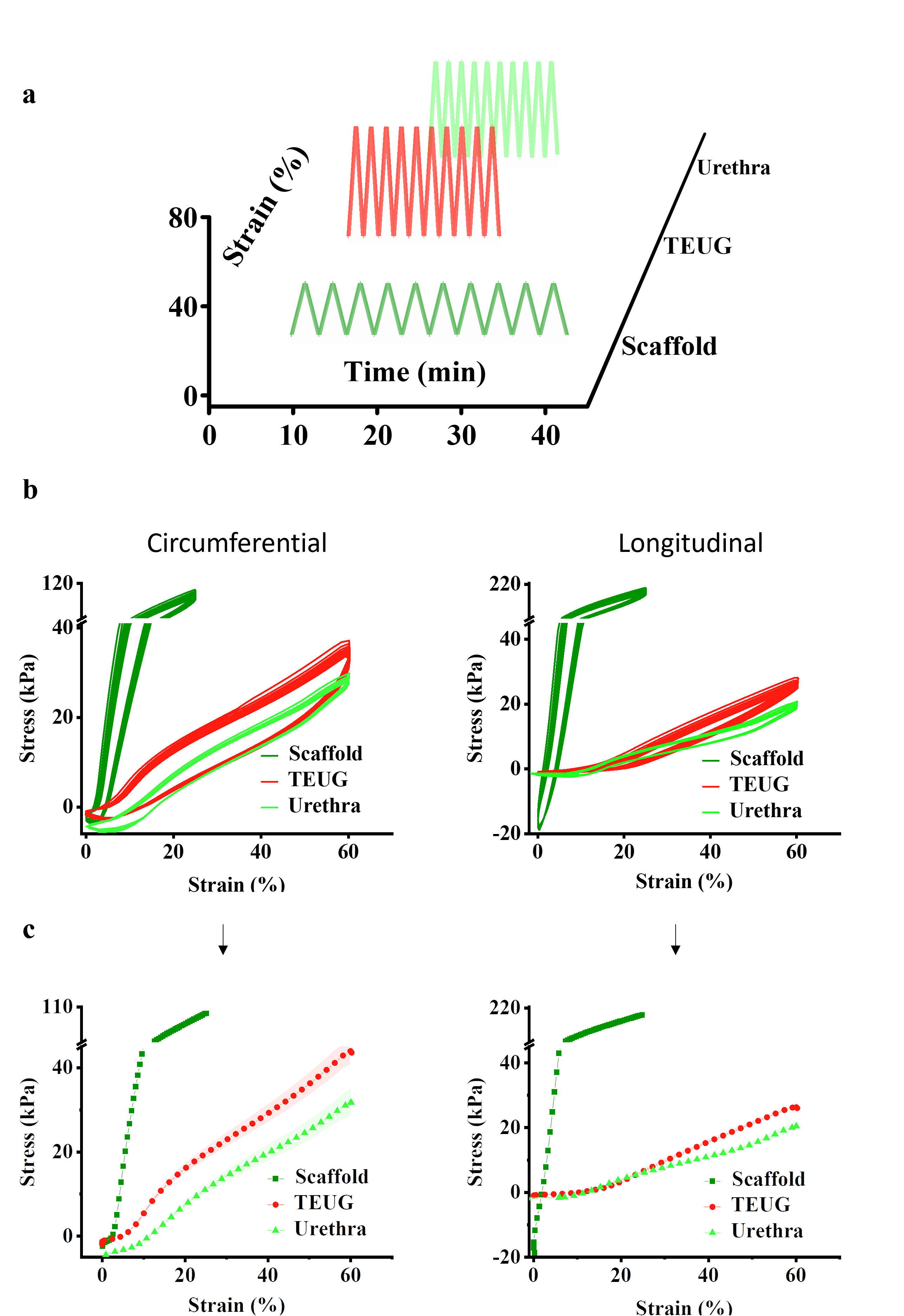


**Figure S5. The elastic response characteristics of the TEUGs constructed in vitro are comparable to those of natural urethras.** a. The 3D waterfall plot intuitively presents the strain-time response curves of three samples during longitudinal tensile loading. b. The circumferential and longitudinal cyclic loading-unloading curves of the three samples at their ultimate tensile strain point. c. The directionally averaged stress-strain curves during the fourth cycle (n = 3). Since the mechanical response curves have stabilized and shown reproducibility after the third cycle, this is used to evaluate the stability of the elastic response of each sample under tensile loading.


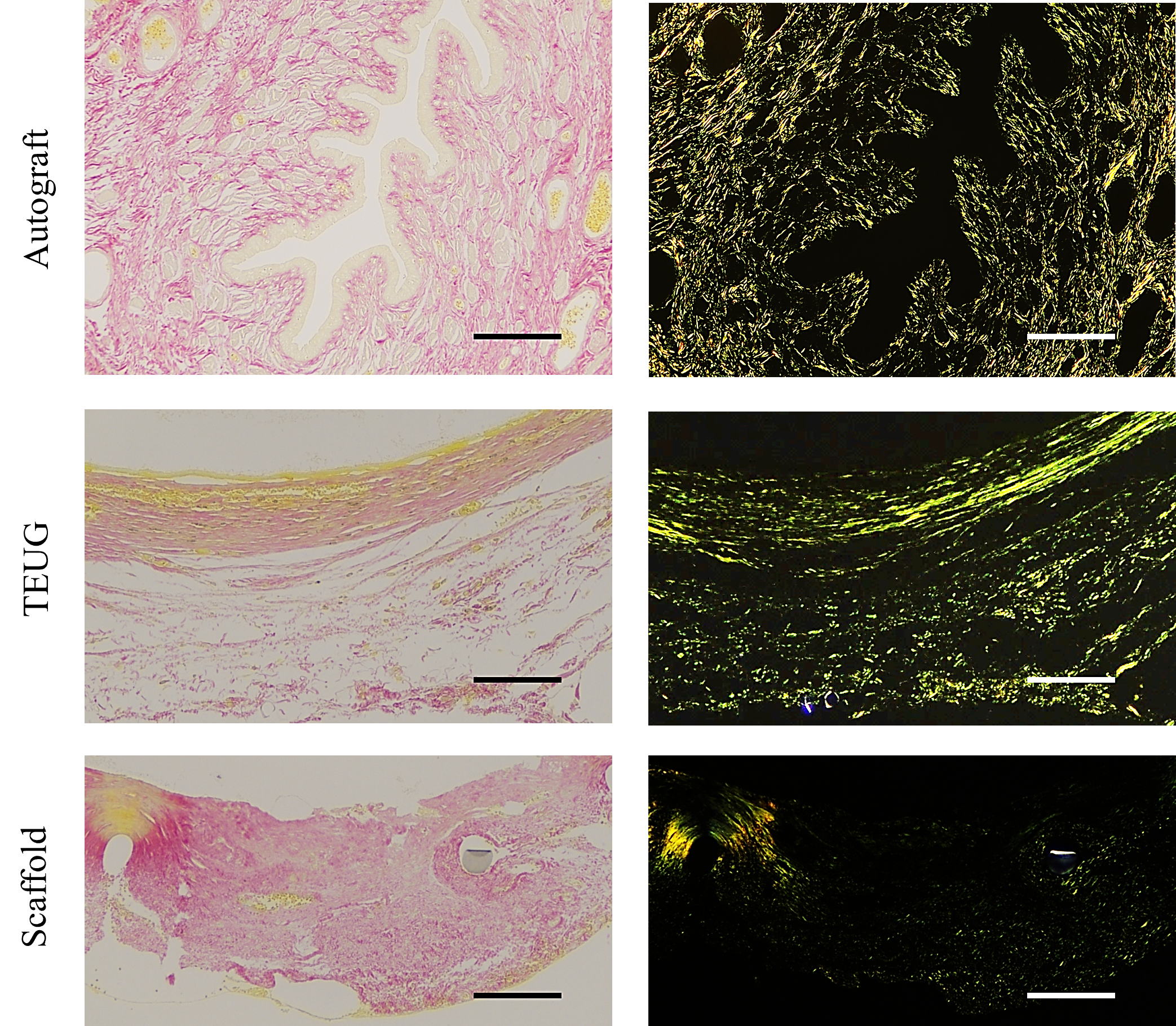


**Figure S6. SR staining of cross-sections of autograft, TEUG, and scaffold tissue sections from retrieved urethral tissues at day 60 post-surgery.** Left panel: light microscope; right panel: polarized microscope. Scale bar, 200 μm.


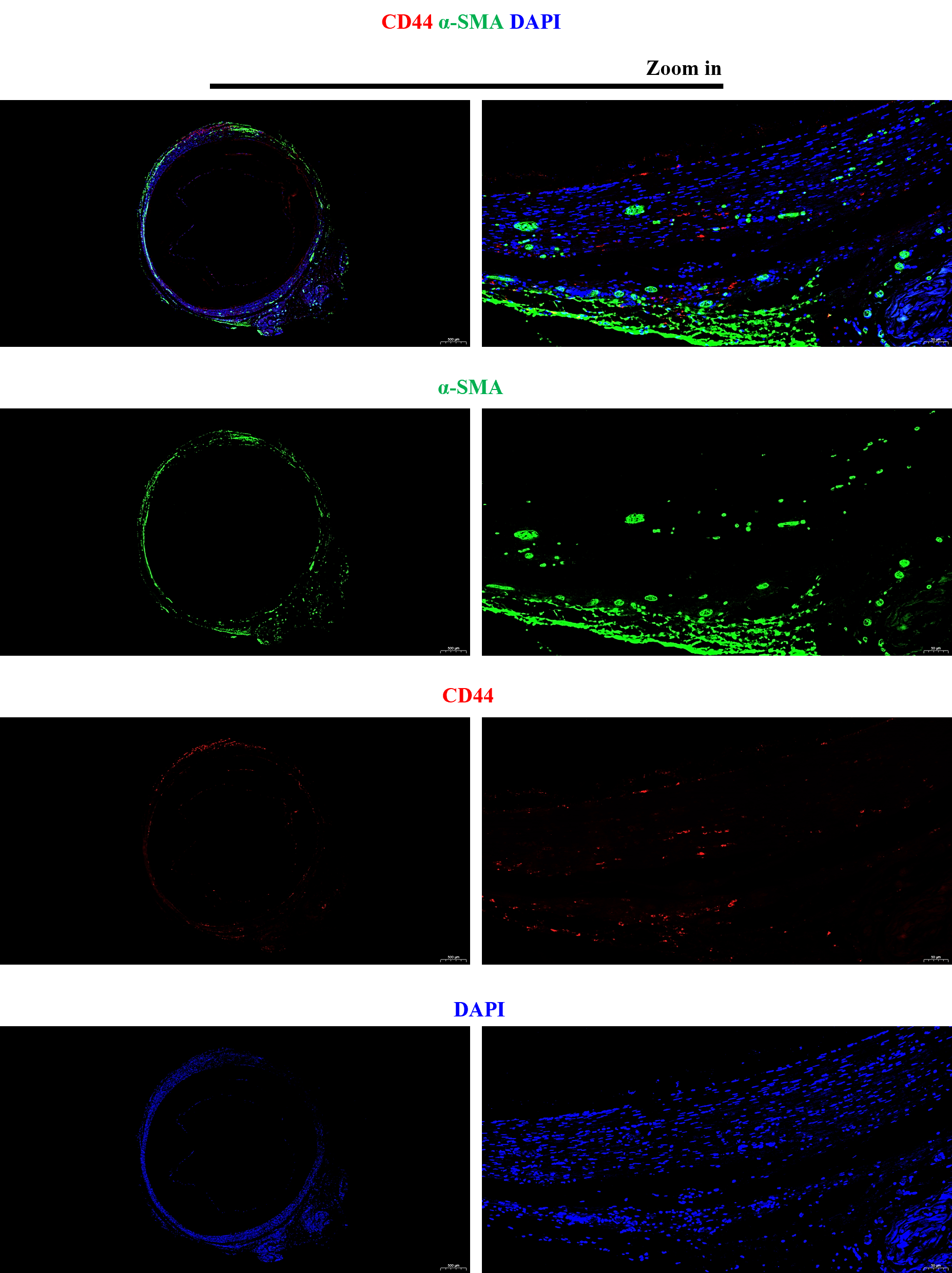


**Figure S7. Immunostaining for CD44 and α-SMA in mid-cross sections of retrieved urethral tissues from TEUG at day 60 post-surgery.** From top to bottom: merged channel, α-SMA channel, CD44 channel, DAPI channel.


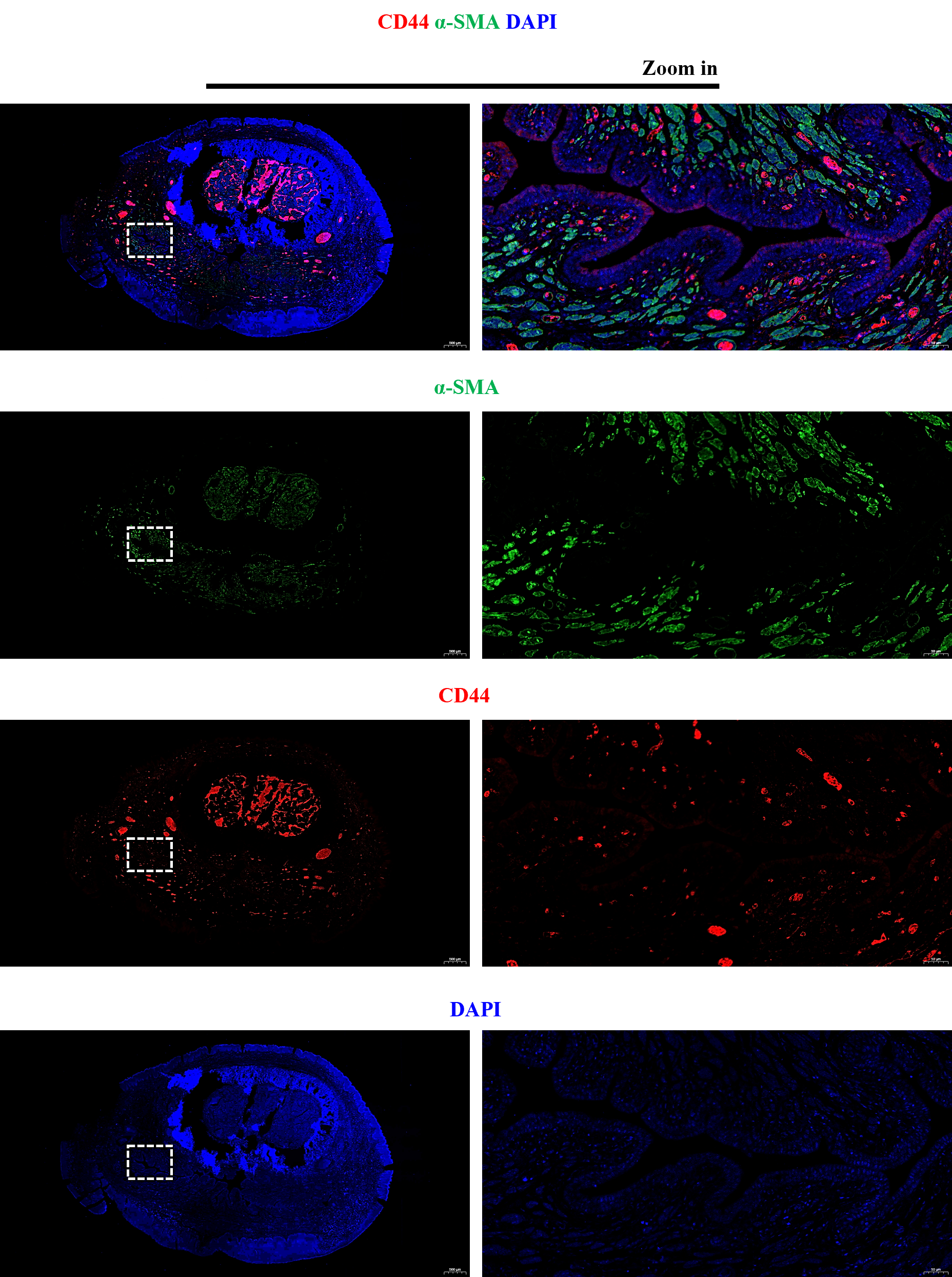


**Figure S8. Immunostaining for CD44 and α-SMA in mid-cross sections of retrieved urethral tissues from autograft at day 60 post-surgery.** From top to bottom: merged channel, α-SMA channel, CD44 channel, DAPI channel.


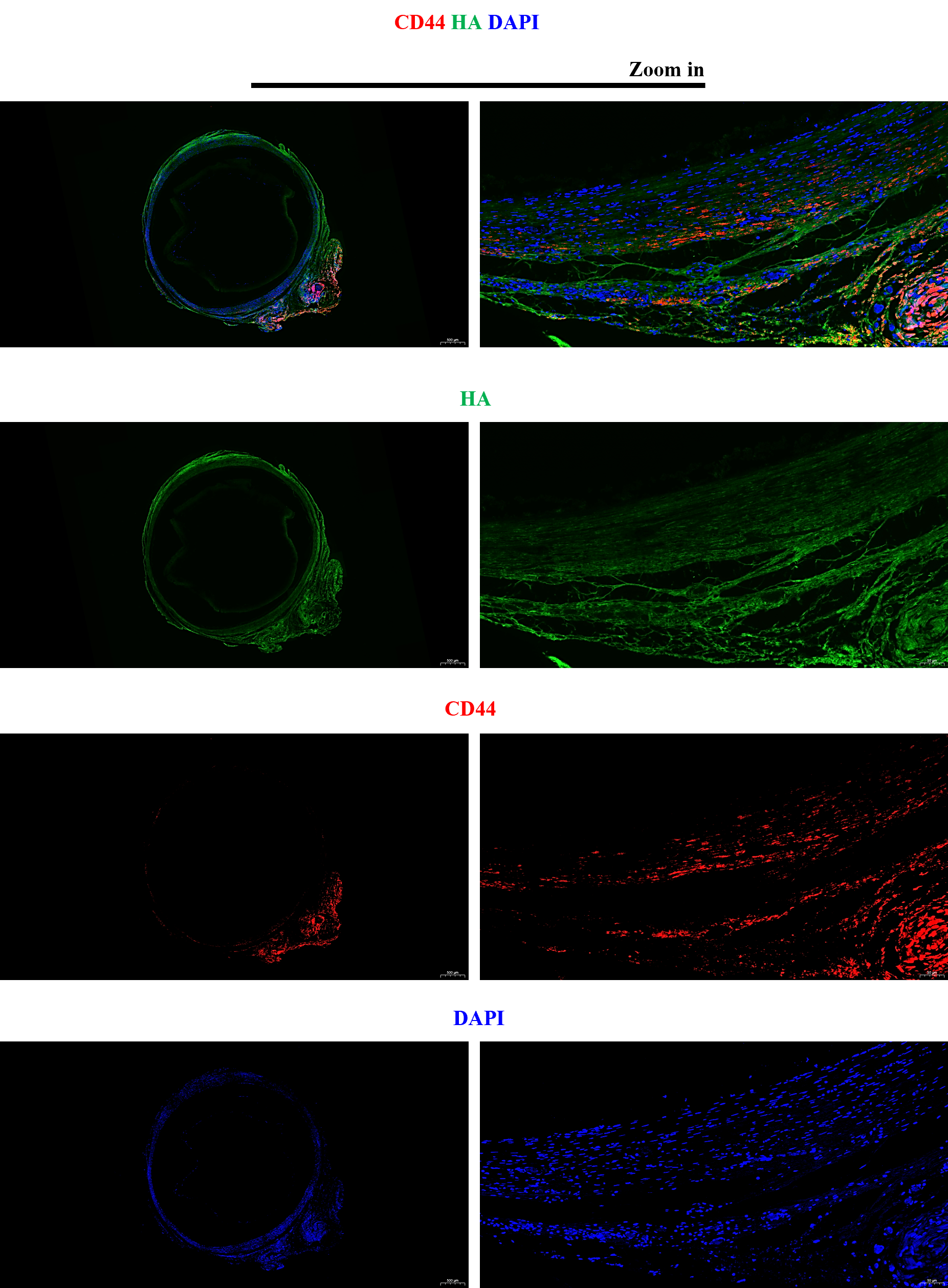


**Figure S9. Immunostaining for CD44 and HA in mid-cross sections of retrieved urethral tissues from TEUG at day 60 post-surgery.** From top to bottom: merged channel, HA channel, CD44 channel, DAPI channel.


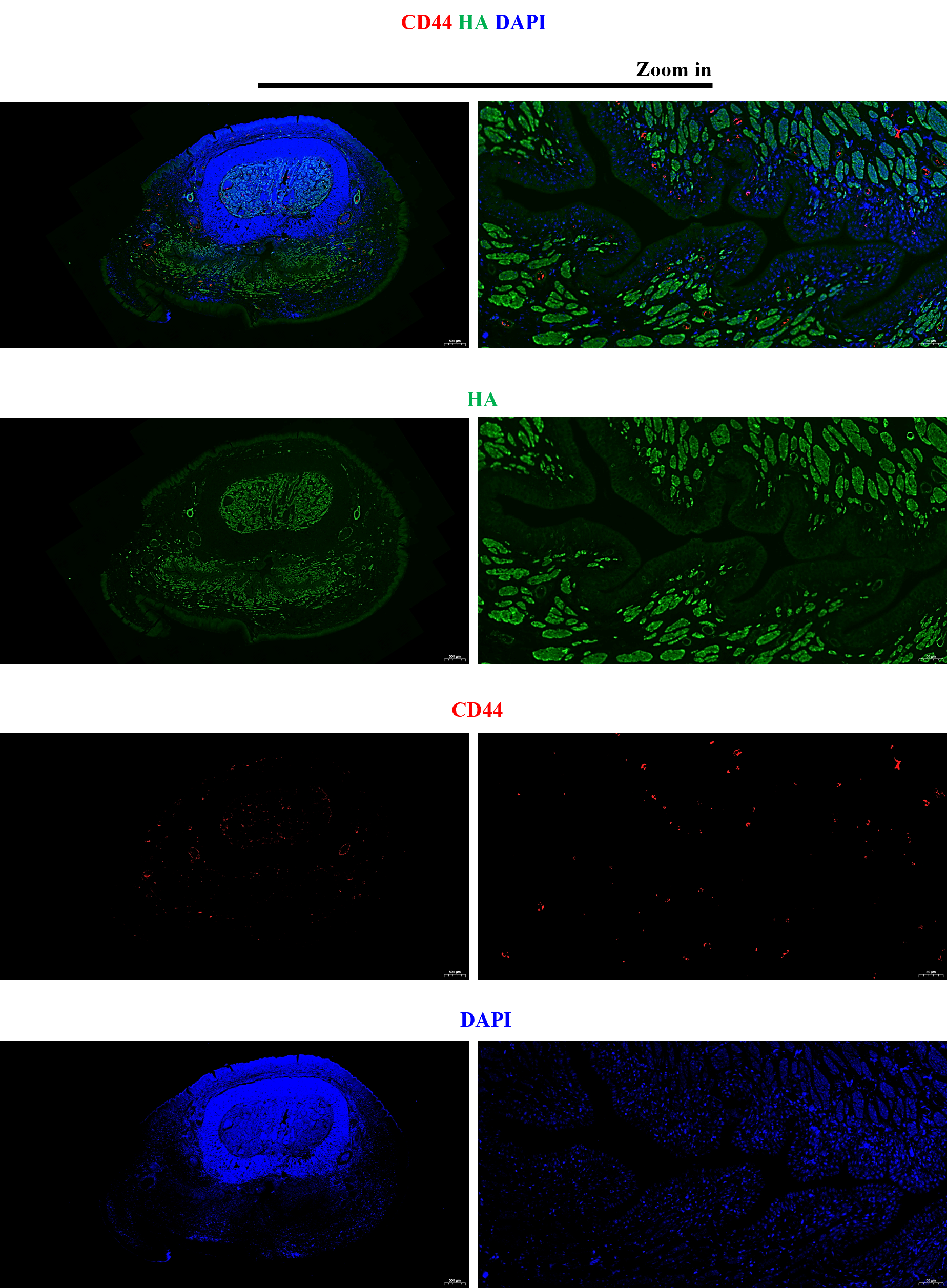


**Figure S10. Immunostaining for CD44 and HA in mid-cross sections of retrieved urethral tissues from autograft at day 60 post-surgery.** From top to bottom: merged channel, HA channel, CD44 channel, DAPI channel.


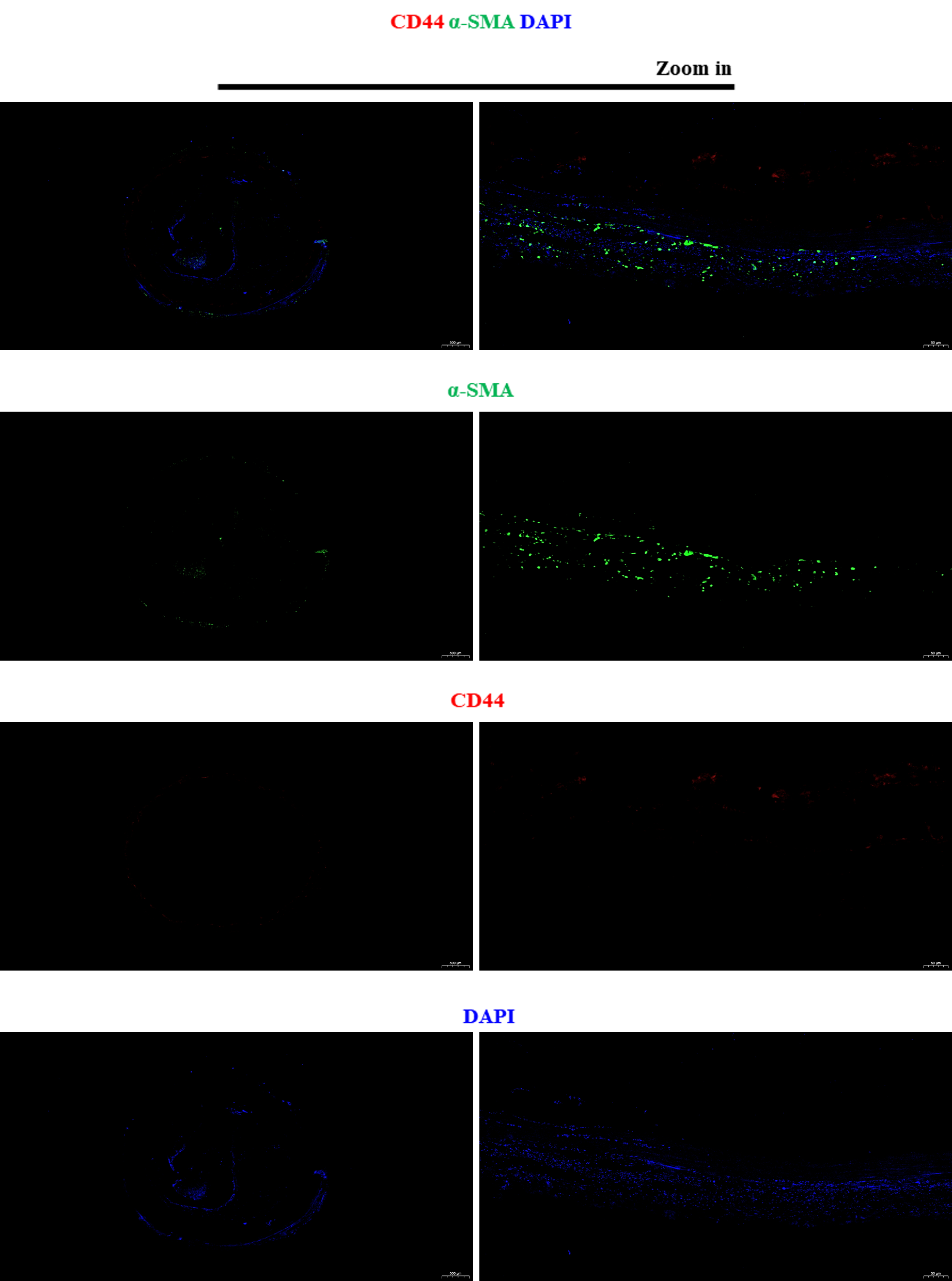


**Figure S11. Immunostaining for CD44 and α-SMA in mid-cross sections of retrieved urethral tissues from scaffold at day 60 post-surgery.** From top to bottom: merged channel, α-SMA channel, CD44 channel, DAPI channel.


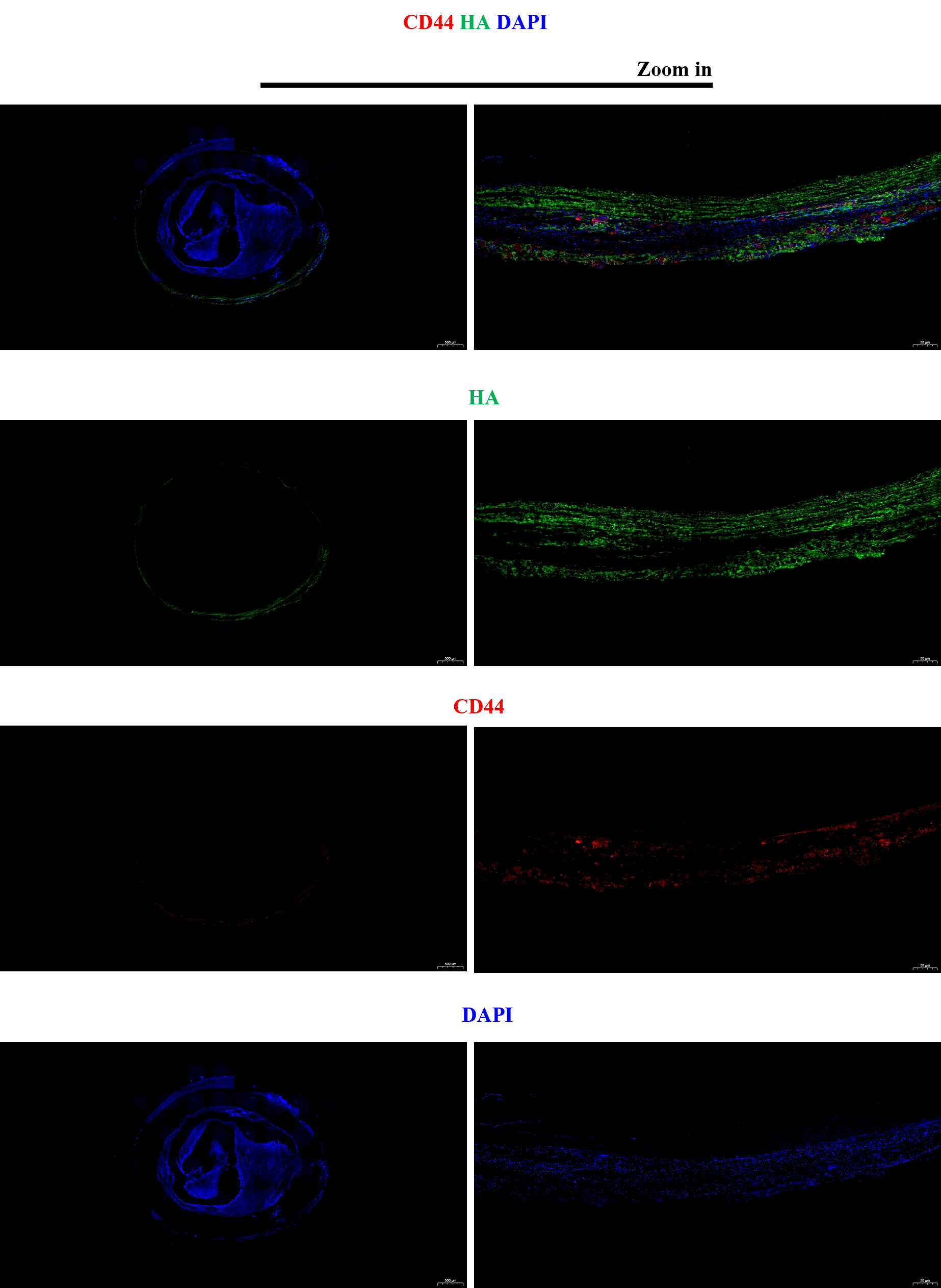


**Figure S12. Immunostaining for CD44 and HA in mid-cross sections of retrieved urethral tissues from scaffold at day 60 post-surgery.** From top to bottom: merged channel, HA channel, CD44 channel, DAPI channel.


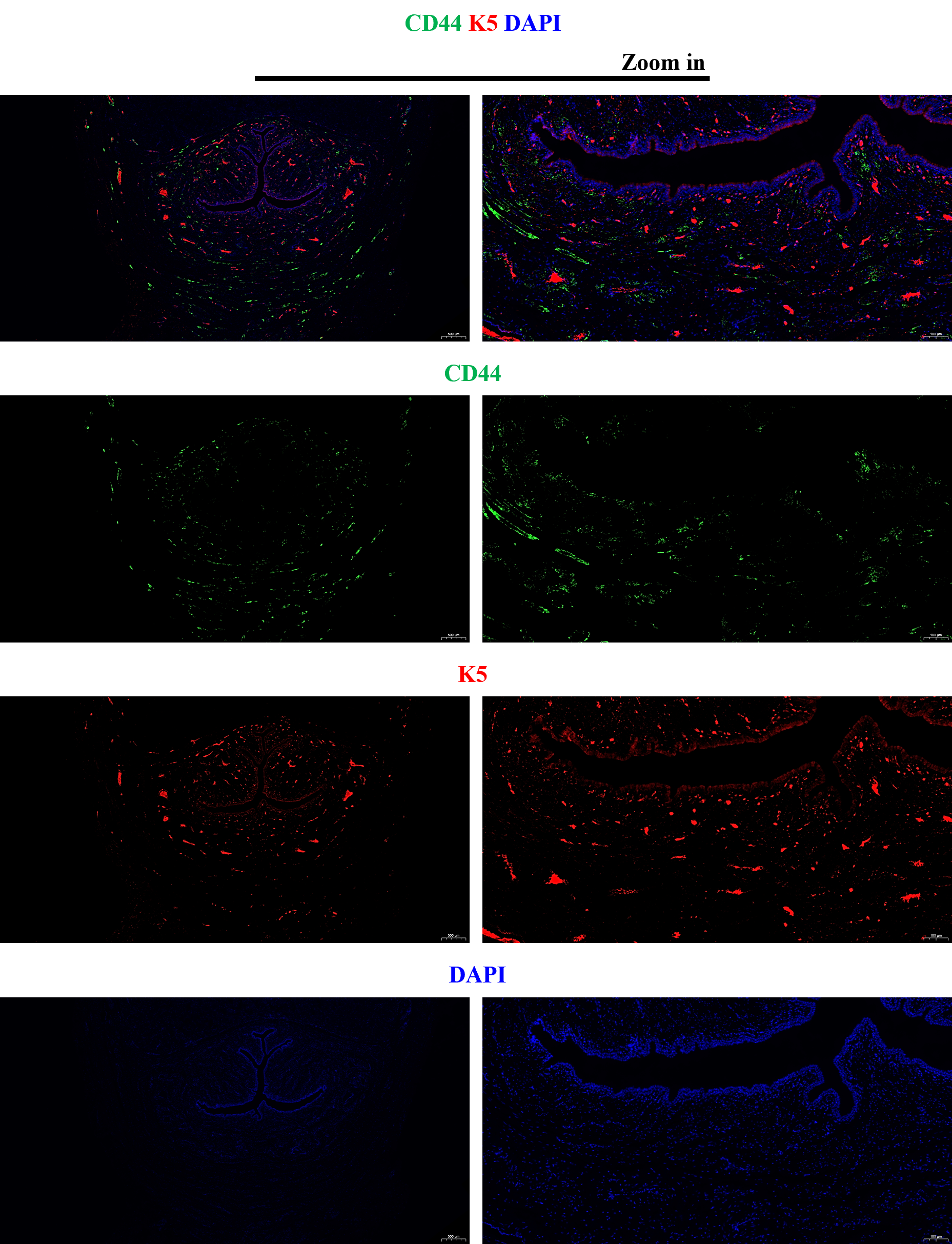


**Figure S13. Immunostaining for CD44 and K5 in mid-cross sections of retrieved urethral tissues from autograft at day 30 post-surgery.** From top to bottom: merged channel, CD44 channel, K5 channel, DAPI channel.


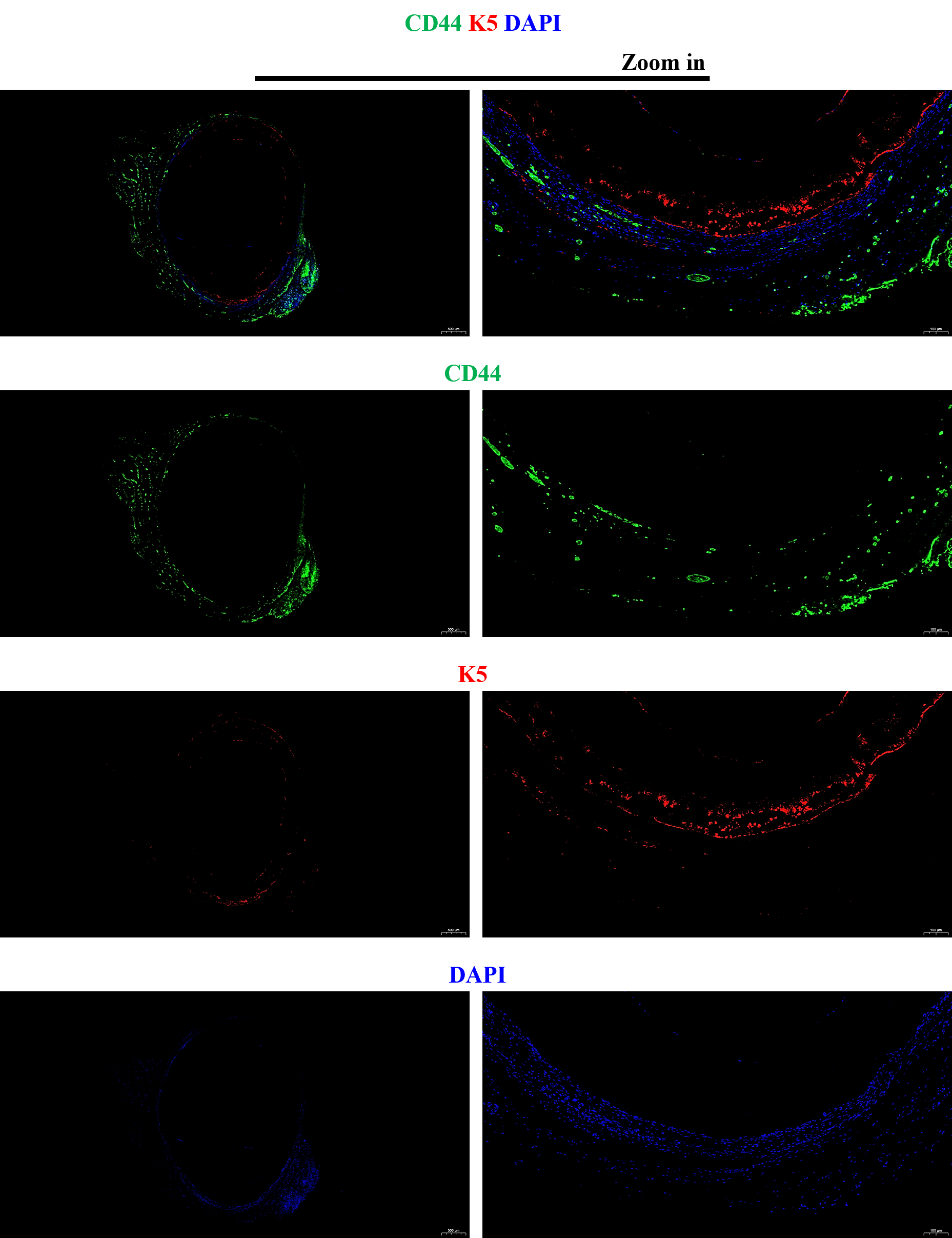


**Figure S14. Immunostaining for CD44 and K5 in mid-cross sections of retrieved urethral tissues from TEUG at day 30 post-surgery.** From top to bottom: merged channel, CD44 channel, K5 channel, DAPI channel.


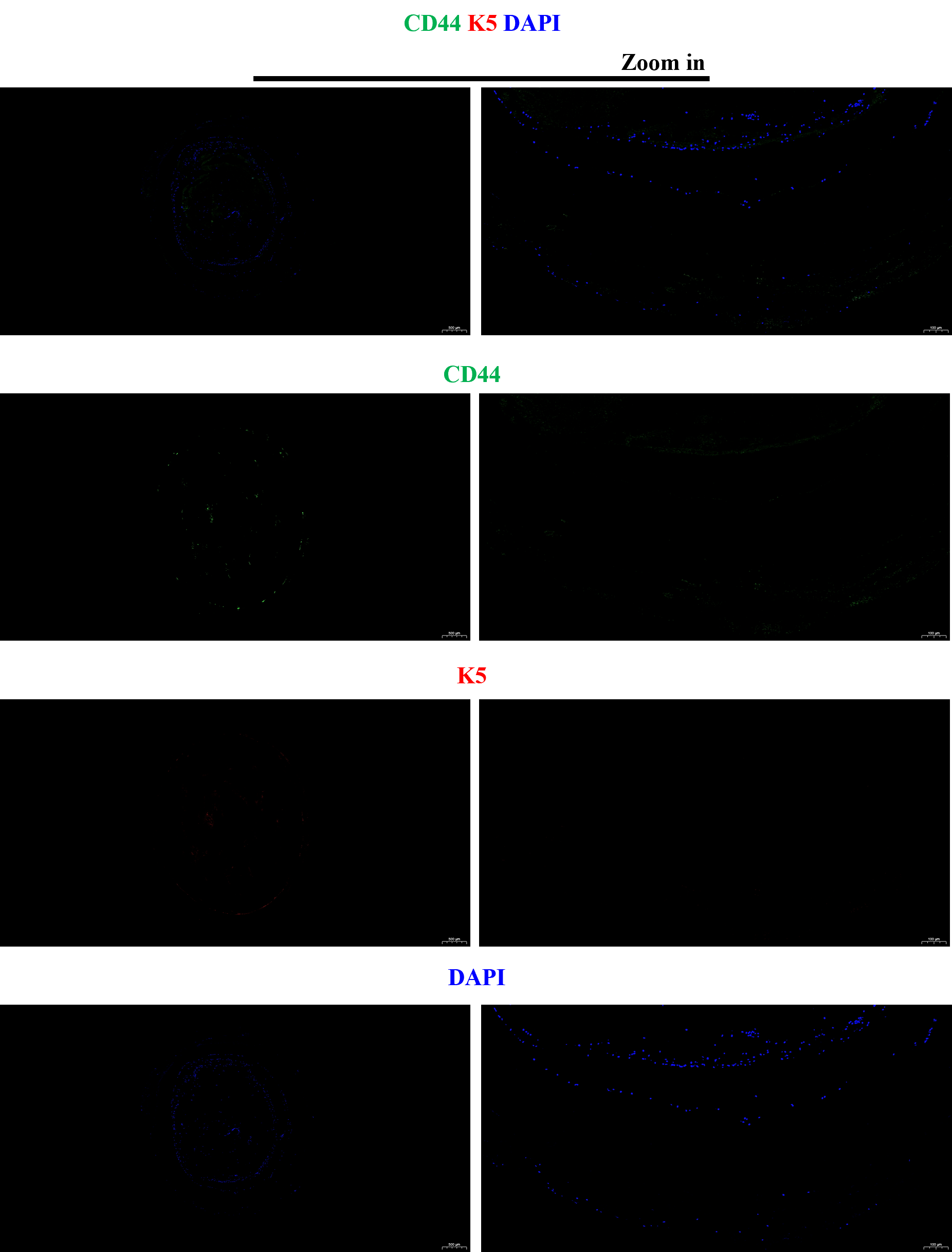


**Figure S15. Immunostaining for CD44 and K5 in mid-cross sections of retrieved urethral tissues from scaffold at day 30 post-surgery.** From top to bottom: merged channel, CD44 channel, K5 channel, DAPI channel.

**Table S1.** Primer sequences used for quantitative RT-PCR analysis.

| **Gene** (rabbit) | **Forward Primer** | **Reverse Primer** |
| --- | --- | --- |
| **GAPDH** | TGAAGGTCGGAGTGAACGGAT | CGTTCTCAGCCTTGACCGTG |
| **TNF-α** | CTCTGCCTCAGCCTCTTCTCTT | ACTTGCGGGTTTGCTACTACG |
| **IL-12** | GTGTCCACGCTTGTCCTTCT | GCTCTCAGCAGGTTTTGGGA |
| **MMP9** | GCTGGCAGAGGCATACTTGTAC | CAGTATGTGATGTTATGATGGTCCC |
| **IL-10** | GCAAGAGGAAGGCGTCTACA | GCAGGTCCCTAACTGATGTCC |
| **TIMP1** | CAGTGTTTCCCTGTTTATCTATCCC | GCAAAGTGACGGCTCTGGTAG |
| **VEGF** | ATGATGAAAGCCTGGAGTGCG | CAAACAAATGCTTTCTCCGCTC |
| **RETNLA** | GGGATGACTGCTACTGGGTG | TCAACGAGTAAGCACAGGCA |
| **ARG1** | TGTCCTTTGCTGATGTCCCTAA | CAGTATGAGCATCCACCCAAAT |
| **CEBPB** | TGGACAAGCTGAGCGACGAG | TTGAACAAGTTCCGCAGGGT |
